# *Anguillicola crassus* infection affects mRNA expression levels in gas gland tissue of European yellow and silver eel

**DOI:** 10.1371/journal.pone.0183128

**Published:** 2017-08-17

**Authors:** Gabriel Schneebauer, Ron P. Dirks, Bernd Pelster

**Affiliations:** 1 Institute of Zoology, University of Innsbruck, Innsbruck, Austria; 2 Center for Molecular Biosciences, University Innsbruck, Innsbruck, Austria; 3 ZF-screens, Leiden, The Netherlands; Xiamen University, CHINA

## Abstract

Using Illumina sequencing, we investigated transcriptional changes caused by the nematode *Anguillicola crassus* within yellow and silver eels by comparing swimbladder samples of uninfected yellow with infected yellow eels, and uninfected silver with infected silver eels, respectively. In yellow eel gas gland, the infection caused a modification of steady state mRNA levels of 1675 genes, most of them being upregulated. Functional annotation analysis based on GO terms was used to categorize identified genes with regard to swimbladder metabolism or response to the infection. In yellow eels, the most prominent category was ‘immune response’, including various inflammatory components, complement proteins, and immunoglobulins. The elevated expression of several glucose and monocarboxylate transporters indicated an attempt to maintain the level of glucose metabolism, even in due to the infection thickened swimbladder tissue. In silver eel swimbladder tissue, on the contrary, the mRNA levels of only 291 genes were affected. Genes in the categories ‘glucose metabolism’ and ‘ROS metabolism’ barely responded to the infection and even the reaction of the immune system was much less pronounced compared to infected yellow eels. However, in the category ‘extracellular matrix’, the mRNA levels of several mucin genes were strongly elevated, suggesting increased mucus production as a defense reaction against the parasite. The present study revealed a strong reaction to an *Anguillicola crassus* infection on mRNA expression levels in swimbladder tissue of yellow eels, whereas in silver eels the changes ware almost negligible. A possible explanation for this difference is that the silvering process requires so much energy that there is not much scope to cope with the additional challenge of a nematode infection. Another possible explanation could be that gas-secreting activity of the silver eel swimbladder was largely reduced, which could coincide with a reduced responsiveness to other challenges, like a nematode infection.

## Introduction

As catadromous fish, European eels *Anguilla anguilla* spend most of their lifetime in European fresh- and coastal water systems as so called yellow eels. After a transformation named silvering, which prepares eels for their long-distance migration and represents the beginning of sexual maturation [1], they return to the species’ expected spawning grounds in the Sargasso Sea for reproduction [2,3]. Because of this complex lifecycle, eels are particularly vulnerable to potential stressors such as overfishing [4], habitat loss [5], pollution [6], changing ocean currents [7], decline of primary production due to increasing sea surface temperature [8], or parasites [9,10]. Almost certain, these stressors somehow act synergistically and have caused a recruitment decline of about 95% since the 1980s [11], resulting in *A*. *anguilla* being listed as critically endangered species by the International Union for the Conservation of Nature and Natural Resources^™^ since 2010 [12].

After eels have passed the continental shelf on their spawning migration, they start performing diel vertical migrations, swimming at depths of 600–1000 m during daytime and 100–300 m during nighttime [13–15]. These daily changes in hydrostatical pressure significantly affect pressure and volume of the swimbladder, functioning as a buoyancy organ [16–19].

During the silvering process, eels not only change body color, their eyes enlarge, neuromasts appear along the lateral line, and body fat content increases [20–22], but also the swimbladder undergoes changes. These changes are thought to improve its gas secreting capacity in order to cope with the significant changes in hydrostatic pressure, encountered during the vertical migrations. Slightly increased wall thickness and vascularization, guanine deposition into the wall to dampen diffusional gas loss and enlargement of the *retia mirabilia* to enhance countercurrent concentration performance [23–25], for example, resulted in a fivefold increase in gas deposition in the American eel *Anguilla rostrata* [23]. The underlying molecular processes of these silvering related improvements and the effects of silvering on various metabolic pathways relevant for swimbladder metabolism, on mRNA level, have been addressed in a recent study [26].

In 1980, the parasitic nematode *Anguillicola crassus* was introduced to Europe by importing infected Japanese eels *Anguilla japonica* from Taiwan to Germany and spread almost throughout the entire eel population within only 10 years [27,28]. Larval stages of the parasite are taken up by the eels via food consumption, invade the swimbladder and, as adults, feed on blood and tissue [27]. This feeding activity and an increasing number of nematodes in the swimbladder lumen, for example, reduce the gas secreting capability of the gas gland cells and swimbladder wall elasticity, and cause various severe pathological changes that can eventually result in loss of swimbladder function [29–31]. The infection with *Anguillicola crassus* has also been shown to impair silvering related improvements in swimbladder function like the ROS defense capacity [32]. In addition, mRNA levels of certain genes, relevant for swimbladder metabolism [26], or the silvering process in general [33] appear to be affected by the nematode infection. However, a comprehensive study on the transcriptional changes in gas gland tissue provoked by the nematode in yellow or in silver eels is missing.

In this study, we therefore investigated the effects of an *Anguillicola crassus* infection on swimbladder tissue at the mRNA level by comparing the swimbladder transcriptome of uninfected yellow eels with infected yellow eels, and of uninfected silver eels with infected silver eels. For comparative reasons, we particularly focused on expression changes related to (1) glucose metabolism and (2) ion exchange, which are required for acid production and release in order to switch on the Root effect for gas secretion [17,18]; (3) angiogenesis, required for appropriate blood supply to the swimbladder [23]; (4) ROS defense, required to avoid oxidative stress related to hyperbaric oxygen tensions [32,34–36]; (5) extracellular matrix, involved in reducing diffusional gas loss from the swimbladder [23–25]; (6) immune response, required to defeat the nematode infection [28,37]; and (7) maturation, which occurs in silver eels during spawning migration [38], because these aspects have been addressed in a previous study, analyzing the transcriptional changes related to silvering [26].

## Materials and methods

### Animals

All experiments were performed with European eels (*Anguilla anguilla*). Uninfected yellow eels were caught by local fishermen in Lake Constance, Bregenz, Austria (N 47° 30’ 54”, E 9° 44’ 35”), and kept in an outdoor freshwater basin at the Institute of Zoology at the University of Innsbruck, until sampling. Infected yellow eels were caught by local fishermen in the River Elbe, close to Winsen (Luhe), Germany (N 53° 24’ 7.7”, E 10° 9’ 27.9”), and kept in an outdoor freshwater basin at the Thünen Institute of Fisheries Ecology, Ahrensburg, Germany, until sampling. All silver eels were caught by local fishermen in the IJsselmeer, The Netherlands (N 52° 49’ 50”, E 5° 25’ 47”), and kept in large tanks at Leiden University until sampling. Recent studies have shown that the European eel is a panmictic species [39,40] and therefore we assumed that the different sampling points should not bias the results of this study. Table 1 shows the morphometrics of the animals, chosen for the experiments, with the silvering index calculated according to Durif et al. [41], and the ocular index calculated according to Pankhurst [42].

**Table 1:** Morphometrics, silvering index according to Durif et al. [41], and ocular index according to Pankhurst [42].

		Uninfected yellow	Infected yellow	Uninfected silver	Infected silver
Body mass	(g)	339.33 ± 7.89	235.60 ± 30.77	1437.36 ± 472.69	830.77 ± 56.21
Body length	(cm)	59.33 ± 1.36	51.80 ± 2.18	82.72 ± 6.08	73.20 ± 2.20
Pectoral fin length	(mm)	23.30 ± 0.46	22.92 ± 1.40	38.08 ± 2.28	36.13 ± 1.11
Horizontal eye diameter	(mm)	5.73 ± 0.35	5.94 ± 0.38	10.42 ± 0.74	10.02 ± 0.34
Vertical eye diameter	(mm)	5.43 ± 0.21	5.80 ± 0.33	10.44 ± 0.70	9.83 ± 0.14
Silvering index		2.00 ± 0.00	2.40 ± 0.22	4.00 ± 0.32	4.17 ± 0.28
Ocular index		4.16 ± 0.41	5.24 ± 0.42	10.35 ± 0.72	10.67 ± 0.62
Number of parasites		0	16.7 ± 3.4	0.4 ± 0.2	11.8 ± 2.5

Uninfected yellow eels (N = 7), infected yellow eels (N = 5), uninfected silver eels (N = 5), and infected silver eels (N = 6). Overall mean values ± S.E.M.

Only swimbladders showing no sign of infection (0 or 1 parasite inside the bladder) or heavily infected swimbladders were selected for the analysis (Table 1). The swimbladder of all infected eels had a similar appearance: thickened, multilayered swimbladder epithelium, exudate inside the bladder, almost no gas filling. We did not include tissue of swimbladders in a transitional state, i.e. with only few nematodes or one or more of the criteria mentioned before (thickened, multilayered swimbladder epithelium; exudate inside the bladder; almost no gas filling) not fulfilled.

### Tissue preparation

Eels were either killed with an overdose of neutralized tricaine methanosulfonate (MS-222; Sigma-Aldrich, St. Luis, MO, USA), or anesthetized with MS-222 and subsequently decerebrated and spinally pithed. The swimbladder was dissected, freed from connective tissue to reveal the actual gas gland tissue, cleaned from *Anguillicola crassus* specimen if necessary, immediately shock frozen in liquid nitrogen, and stored at -80°C until further use. Infected swimbladders contained between 5 and 30 parasites, and the swimbladder wall was markedly thickened and nontransparent as stated previously [30]. Tissue sampling was performed in compliance with the Austrian law, the guidelines of the Austrian Federal Minister for Education, Arts, and Culture, and also the Dutch and German law. The tissue sampling procedure was approved by the Tierversuchskommission of the University of Innsbruck.

### RNA isolation and Illumina RNASeq analysis

Total RNA was isolated from gas gland tissue using the Qiagen miRNeasy kit (Qiagen, Venlo, Netherlands) as established and described in detail in a previous study [26]. Briefly, quality and integrity of the isolated RNA were checked on an Agilent Bioanalyzer 2100 total RNA Nano series II chip (Agilent, Amstelveen, Netherlands). Illumina RNAseq libraries were prepared from 2 μg total RNA using the Illumina TruSeq^™^ RNA Sample Prep Kit v2 according to the manufacturer’s instructions (Illumina Inc. San Diego, CA, USA). All RNAseq libraries (150–750 bp inserts) were sequenced on an Illumina HiSeq2500 sequencer as 2 × 50 nucleotides paired-end reads according to the manufacturer’s protocol. Image analysis and base calling were done using the Illumina pipeline [43,44]. The data discussed in this publication have been deposited in NCBI's Gene Expression Omnibus and are accessible through GEO Series accession number GSE102221 (https://www.ncbi.nlm.nih.gov/geo/query/acc.cgi?acc=GSE102221).

### Illumina data processing

Data processing was performed as described previously [26,43,44]. Briefly, reads (10–20 million per sample) were aligned to the draft genome sequence of European eel [45], using TopHat (version 2.0.5) [46]. Secondary alignments of reads were excluded by filtering the files using SAMtools (version 0.1.18) [47]. Aligned fragments per predicted gene were counted from SAM alignment files using the Python package HTSeq (version 0.5.3p9) [48]. In order to make comparisons across samples possible, these fragment counts were corrected for the total amount of sequencing performed for each sample. As a correction scaling factor, library size estimates determined using the R/Bioconductor (release 2.11) package DESeq [49] were employed. Read counts were normalized by dividing the raw counts obtained from HTSeq by its scale factor. Detailed read coverage for individual genes was extracted from the TopHat alignments using SAMtools. Differentially expressed genes between uninfected yellow and infected yellow eels and also between uninfected silver and infected silver eels were identified using DESeq, the cut-off for significance was set to P<0.01. Gene ontology annotations were used for a detailed pathway and biological process analysis of differentially expressed genes.

## Results

### General observations

**Fig 1:**
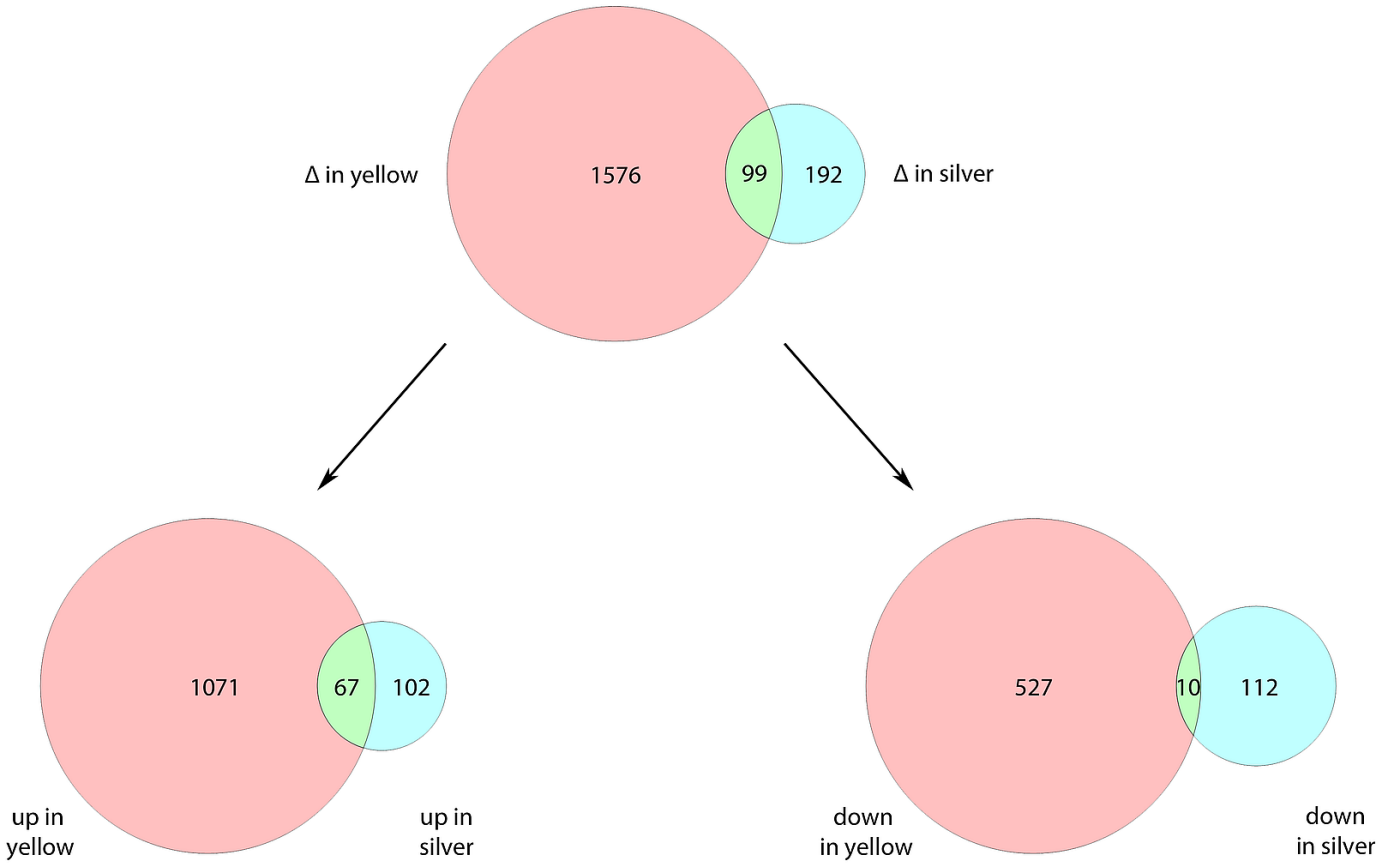
Unequally severe impact of an *Anguillicola crassus* infection on gene transcription. Venn diagrams showing the total numbers of differentially transcribed genes in yellow eel (red) and silver eel (blue) gas gland tissue due to the infection with *Anguillicola crassus*, and the number of genes affected in both groups (green). The lower part shows the numbers of genes either up- or downregulated. Diagrams were generated with Venn Diagram Plotter (https://omics.pnl.gov/software/venn-diagram-plotter).

**Fig 2:**
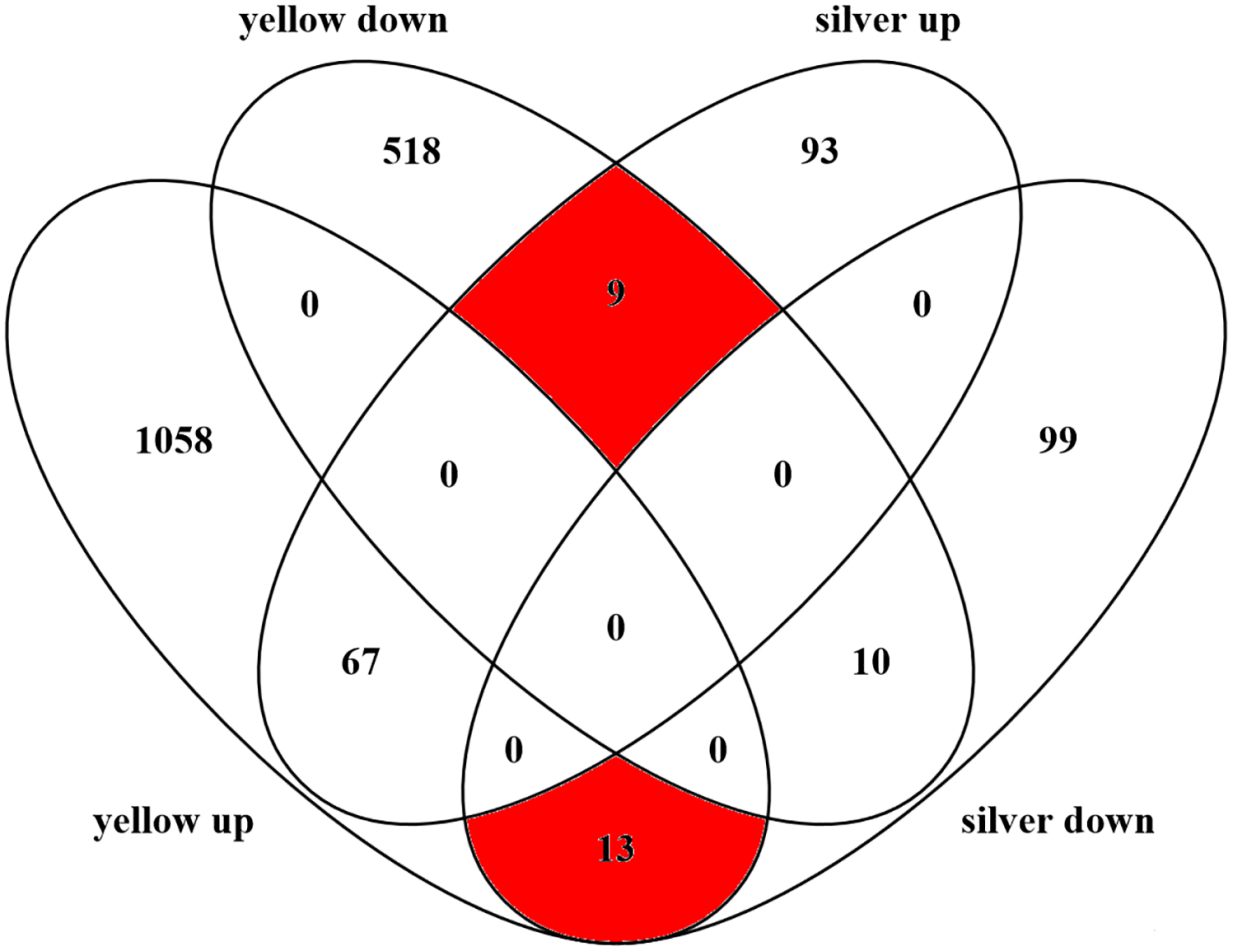
Contradictorily affected genes transcription. Venn diagram showing the total numbers of up- or downregulated genes, caused by an infection with *Anguillicola crassus*, in yellow and silver eel gas gland tissue with special emphasis on the number of genes (red), which were upregulated in one group but simultaneously downregulated in the other group. Diagram was generated with Venny 2.1.0 (http://bioinfogp.cnb.csic.es/tools/venny/index.html).

Comparing uninfected and infected yellow and silvers eels, even at a significance level of p<0.01 a large number of genes showed different expression levels, especially in yellow eels. In yellow eel gas gland tissue, an *Anguillicola crassus* infection resulted in 1675 differentially transcribed genes of which 1138 were upregulated and 537 were downregulated. In silver eels, the infection resulted in only 291 differentially transcribed genes of which 169 were upregulated and 122 were downregulated (Fig 1). Ninety-nine genes were transcribed differentially in yellow eels as well as in silver eels, of which 67 were upregulated and 10 were downregulated in infected yellow eels as well as in infected silver eels. Twenty-two genes were differently affected in infected yellow and silver eels (Fig 2; Table 2). Thirteen of these genes were upregulated in yellow eels but downregulated in silver eels, and 9 genes were downregulated in yellow eels but upregulated in silver eels.

**Table 2:** Differentially transcribed and contradictorily regulated genes in infected yellow and infected silver eels as compared with uninfected yellow and uninfected silver eels, respectively.

Gene	Name	Description	Yellow		Silver	
			Fold cha.	pval	Fold cha.	pval
g26738	hfe	hereditary hemochromatosis protein	0.41	0.004	24.64	0.000
g24844	rergl	ras-related and estrogen-regulated growth inhibitor-like protein	0.41	0.006	4.38	0.003
g11737	noxo1	nadph oxidase organizer 1	0.38	0.008	15.96	0.000
g2232	un13c	protein unc-13 homolog c	0.35	0.008	7.78	0.006
g27646	c2c4c	c2 calcium-dependent domain-containing protein 4c	0.26	0.000	40.43	0.000
g28445	st17a	serine threonine-protein kinase 17a	0.22	0.007	9.58	0.004
g16142	ticn1	testican-1	0.21	0.000	16.89	0.000
g11645	neca1	n-terminal ef-hand calcium-binding protein 1	0.15	0.000	13.17	0.000
g17980	irg1	immune-responsive gene 1 protein	0.04	0.000	3.66	0.002
g14663	cp1b1	cytochrome p450 1b1	11.08	0.000	0.22	0.002
g8112	rimb2	rims-binding protein 2	9.59	0.000	0.25	0.006
g5564	s39ac	zinc transporter zip12	7.17	0.000	0.22	0.006
g28431	mlp3c	microtubule-associated proteins 1a 1b light chain 3c	5.25	0.000	0.03	0.000
g12939	cdkn3	cyclin-dependent kinase inhibitor 3	4.49	0.000	0.09	0.009
g26398	nptx1	neuronal pentraxin-1	3.88	0.004	0.09	0.000
g850	degs2	sphingolipid delta -desaturase c4-hydroxylase des2	3.42	0.002	0.15	0.006
g14690	cpas1	circularly permutated ras protein 1 1	2.84	0.000	0.24	0.002
g26753	cxl11	c-x-c motif chemokine 11	2.71	0.000	0.17	0.000
g9811	ctl2b	protein ctla-2-beta	2.55	0.000	0.13	0.003
g18466	akr	homeobox protein akr	2.49	0.007	0.11	0.000
g9993	a33	zinc-binding protein a33	2.44	0.000	0.26	0.004
g20093	pclo	protein piccolo	2.13	0.001	0.23	0.002

Fold cha. = Fold change; pval = 0.000 indicates P values < 0.0005

Elevated in infected silver eels, but expressed at a lower level in infected yellow eels were NADPH oxidase oxygenizer 1 (noxo1) and two Ca^2+^ binding proteins, C2 calcium-dependent domain containing protein 4c (c2c4c) and the ef-hand calcium-binding protein 1 (neca1) (Table 2). Elevated in infected yellow eels but reduced in infected silver eel gas gland tissue were cytochrome P4501b1 (cp1b1), and two zinc binding proteins, zinc transporter zip112 (s39ac) and zinc binding protein a33 (a33). In addition, c-x-c motif chemokine 11 (cxl11) was elevated 2.71-fold, while it was 5.88-fold reduced in infected silver eel gas gland tissue.

**Fig 3:**
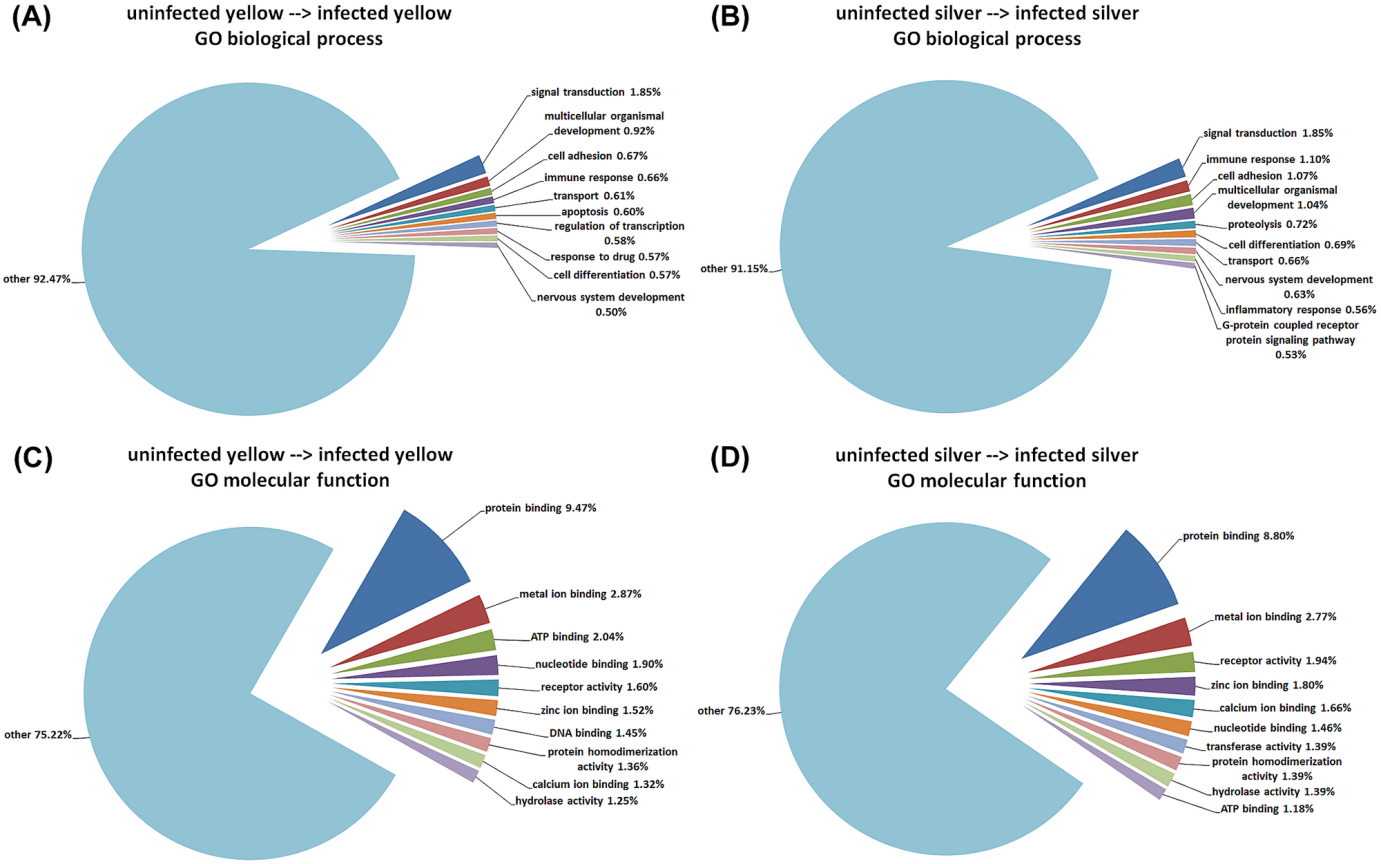
Most important targets of an *Anguillicola crassus* infection. The ten most prominent biological processes, affected by the infection with *Anguillicola crassus* in yellow (A) and silver eel (B) gas gland tissue, respectively. The ten most prominent molecular functions, affected by the infection with *Anguillicola crassus* in yellow (C) and silver eel (D) gas gland tissue, respectively.

Fig 3 shows the results of a GO enrichment analysis for GO biological processes and GO molecular function, focusing on the 10 categories with the largest number of hits, and combining the remaining genes as ‘others’. With respect to biological processes, a very large number of diverse processes showed a number of genes with modified expression levels, so that in infected yellow and silver eels 92.5% and 91.2% of the modified genes, respectively, were combined as ‘others’. Processes affected in both, infected yellow and silver eels, included ‘signal transduction’, ‘multicellular organismal development’, ‘immune response’, ‘cell adhesion’, ‘transport’, ‘cell differentiation’, and ‘nervous system development’. Processes included in the 10 categories with a larger number of hits in infected yellow eels, but not in silver eels, were ‘apoptosis’, ‘regulation of transcription’, and ‘response to drug’. In infected silver eels, in turn, ‘proteolysis’, ‘inflammatory response’, and ‘G-protein coupled receptor protein signaling pathway’ were among the 10 categories with a larger number of hits. The same analysis for GO molecular function revealed less diversity, and 75.2% and 76.2% of the genes were listed as ‘others’ in infected yellow and infected silver eels, respectively. The molecular function with the largest number of hits was ‘protein binding’, contributing 9.5% and 8.8% to the total number of modified genes in infected yellow and infected silver eels, respectively. ‘DNA binding’ was among the 10 categories with the largest number of hits in infected yellow eels, but not in silver eels, and ‘transferase activity’ was among the 10 top categories in infected silver eels, but not in yellow eels.

### Transcriptional changes in yellow eel gas gland tissue related to the nematode infection

As the next step, we performed the GO enrichment analysis focusing on genes of specific functional categories expected to be important for swimbladder function, i.e. glucose and lactate metabolism, ROS defense, ion transport, extracellular matrix, and vasculogenesis and angiogenesis. We also included immune defense and maturation, which have been reported to be important categories in a previous study [26]. Especially in yellow eels, a large number of genes were affected in the expression level. We therefore restricted our analysis to genes showing at least a 3-fold difference in the mRNA expression level.

#### Glucose and lactate metabolism

In gas gland tissue of infected yellow eels, 4 genes involved in monocarboxylate transport and glucose transport showed a significantly higher mRNA expression level than in uninfected yellow eels (Table 3). In addition, the mRNA level of fructose-bisphosphate aldolase A increased 5.66-fold, while the glucokinase mRNA level decreased 12.5-fold.

**Table 3:** Differentially transcribed genes (fold change >3) based on GO terms “glucose metabolism” or “lactate metabolism” in infected yellow and infected silver eels as compared with uninfected yellow and uninfected silver eels, respectively.

Gene	Name	Description	Yellow		Silver	
			Fold cha.	pval	Fold cha.	pval
g42062	sc5a8	sodium-coupled monocarboxylate transporter 1	Inf	0.000		
g14954	sc5a8	sodium-coupled monocarboxylate transporter 1	18.00	0.002		
g21440	aldoa	fructose-bisphosphate aldolase a	5.66	0.000		
g13449	gtr5	solute carrier family facilitated glucose transporter member 5	5.12	0.000		
g42042	tec	tyrosine-protein kinase tec	4.44	0.006		
g27889	fyn	tyrosine-protein kinase fyn	3.46	0.000		
g23774	ppara	peroxisome proliferator-activated receptor alpha	0.31	0.005		
g21936	npas4	neuronal pas domain-containing protein 4	0.29	0.003		
g3113	hxk4	glucokinase	0.08	0.009		
g12848	gtr3	solute carrier family facilitated glucose transporter member 3	3.18	0.000	6.18	0.003
g7770	mt12b	monocarboxylate transporter 12-b			4.91	0.006
g22031	acs2l	acetyl-coenzyme a synthetase 2- mitochondrial			4.37	0.007
g21839	k6pf	6- muscle type			0.14	0.008

Fold cha. = Fold change; pval = 0.000 indicates P values < 0.0005

#### ROS defense

Also important for swimbladder function is ROS defense to avoid tissue damage due to high oxygen partial pressures, and 40 genes related to ROS were affected in their mRNA expression level in infected yellow eels (Table 4). The expression level of several transcription factors was significantly increased (fosb; fos; junb), and at least two copies of each of these transcription factors were affected. The expression level of one copy of fos and one of fosb was elevated more than 20-fold. Cytochrome b-245 heavy chain (cy24b) and cytochrome p450 1b1 (cp1b1) were found with elevated expression levels.

**Table 4:** Differentially transcribed genes (fold change >3) based on GO terms related to “ROS defense” in infected yellow and infected silver eels as compared with uninfected yellow and uninfected silver eels, respectively.

Gene	Name	Description	Yellow		Silver	
			Fold cha.	pval	Fold cha.	pval
g38341	angl7	angiopoietin-related protein 7	Inf	0.004		
g11898	fosb	protein fosb	21.55	0.000		
g12410	fos	proto-oncogene c-fos	20.06	0.000		
g24314	dscam	down syndrome cell adhesion molecule homolog flags: precursor	11.14	0.005		
g5407	hspbb	heat shock protein beta-11	10.00	0.000		
g6637	cy24b	cytochrome b-245 heavy chain	9.01	0.001		
g10399	plcz1	1-phosphatidylinositol- -bisphosphate phosphodiesterase zeta-1	8.85	0.003		
g16623	tutlb	protein turtle homolog b	5.62	0.004		
g5642	dus2	dual specificity protein phosphatase 2	5.15	0.000		
g12409	fos	proto-oncogene c-fos	4.96	0.000		
g5410	hspbb	heat shock protein beta-11	4.84	0.000		
g12220	rir2	ribonucleoside-diphosphate reductase subunit m2	4.78	0.000		
g11400	junb	transcription factor jun-b	4.20	0.000		
g6522	trpa1	transient receptor potential cation channel subfamily a member 1	4.13	0.005		
g3425	plcb2	1-phosphatidylinositol- -bisphosphate phosphodiesterase beta-2	4.08	0.002		
g12361	junb	transcription factor jun-b	4.06	0.000		
g7816	hmox	heme oxygenase	4.05	0.000		
g17581	lox5	arachidonate 5-lipoxygenase	3.86	0.001		
g3322	fos	proto-oncogene c-fos	3.70	0.000		
g15583	nud17	nucleoside diphosphate-linked moiety x motif 17	3.38	0.000		
g17446	paxi	Paxillin	3.35	0.000		
g12497	kpcb	protein kinase c beta type	3.34	0.004		
g10644	arrd4	arrestin domain-containing protein 4	3.26	0.000		
g5757	plcg2	1-phosphatidylinositol- bisphosphate phosphodiesterase gamma-2	3.23	0.000		
g4039	cdk1	cell division protein kinase 1	3.19	0.000		
g24609	kcc1d	calcium calmodulin-dependent protein kinase type 1d	3.19	0.001		
g14921	ets1a	protein c-ets-1-a	3.11	0.003		
g7735	cy24b	cytochrome b-245 heavy chain	3.07	0.000		
g28565	rir2	ribonucleoside-diphosphate reductase subunit m2	3.03	0.005		
g7976	adam9	disintegrin and metalloproteinase domain-containing protein 9	0.33	0.001		
g26034	cp27a	sterol 26- mitochondrial	0.32	0.000		
g21936	npas4	neuronal pas domain-containing protein	0.29	0.003		
g17084	achb2	neuronal acetylcholine receptor subunit beta-2	0.24	0.005		
g14480	myh7	myosin-7	0.16	0.000		
g2246	mk10	mitogen-activated protein kinase 10	0.14	0.000		
g2030	pa24c	cytosolic phospholipase a2 gamma	0.12	0.000		
g21973	nptx1	neuronal pentraxin-1	0.07	0.000		
g14663	cp1b1	cytochrome p450 1b1	11.08	0.000	0.22	0.002
g26398	nptx1	neuronal pentraxin-1	3.88	0.004	0.09	0.000
g12711	mmp9	matrix metalloproteinase-9	3.64	0.000	23.32	0.000
g26738	hfe	hereditary hemochromatosis protein			24.64	0.000
g19822	co5a1	collagen alpha-1 chain flags: precursor			0.33	0.005
g24694	mmp17	matrix metalloproteinase-17			0.08	0.000

Fold cha. = Fold change; pval = 0.000 indicates P values < 0.0005

#### Ion transport

With respect to ion transport, 56 genes showed modified expression levels in infected yellow eels, and only 18 of these were reduced (S1 Table). In addition to monocarboxylate transporter 1, which was present at very high levels in infected yellow eel gas gland tissue, two amino acid transporters were elevated almost 4-fold (y+1 amino acid transporter 2, ylat2; and sodium-dependent neutral amino acid transporter b at1; s6a19). In infected yellow eel gas gland tissue, a large number of Na^+^, K^+^, or Cl^-^ transporting proteins were expressed with significantly modified mRNA levels: orphan sodium and chloride-dependent neurotransmitter transporter ntt73, s6a15; voltage-dependent anion-selective channel protein 2, vdac2; transient receptor potential cation channel subfamily a member 1, trpa1; solute carrier family 12 member 2, s12a2; electrogenic sodium bicarbonate cotransporter 1, s4a4; potassium voltage-gated channel subfamily c member 1, kcnc1; chloride channel protein 2, clcn2; calcium-activated potassium channel subunit alpha-1, kcma1; solute carrier family 12 member 5, s12a5; cystic fibrosis transmembrane conductance regulator, cftr; sodium channel protein type 5 subunit alpha, scn5a; amiloride-sensitive cation channel neuronal, accn1; solute carrier family 13 member 3, s13a3. Seven of these genes showed an increased expression level, while 6 of these transporters, like cftr, clcn2, and s12a5, showed a reduced expression level. Interestingly, sodium potassium-transporting atpase subunit beta-2 (at1b2) also showed a more than 8-fold reduction in the expression level.

#### Extracellular matrix

The mRNA expression level of 11 genes was modified in infected yellow eel gas gland tissue, and all but one were elevated (Table 5). Connective tissue growth factor (ctgf) was more than 5-fold elevated, and the level of collagen alpha 6 (co6a6) and versican core protein (cspg2) was increased. Acidic mammalian chitinase (chia) was 5-6-fold elevated. Similarly, thrombospondin-1 (tsp1) and thrombospondin 4b (tsp4b) were almost 5-fold elevated. Of the various mucin genes only mucin 5ac (muc5a) was 3-fold elevated.

**Table 5:** Differentially transcribed genes (fold change >3) based on GO terms related to “extracellular matrix” in infected yellow and infected silver eels as compared with uninfected yellow and uninfected silver eels, respectively.

Gene	Name	Description	Yellow		Silver	
			Fold cha.	pval	Fold cha.	pval
g16623	tutlb	protein turtle homolog b	5.62	0.004		
g22618	chia	acidic mammalian chitinase	5.05	0.000		
g7750	tsp4b	thrombospondin-4-b	4.97	0.000		
g1692	tsp1	thrombospondin-1 flags: precursor	4.84	0.000		
g14358	co6a6	collagen alpha-6 chain flags: precursor	3.40	0.000		
g5094	scub3	signal cub and egf-like domain-containing protein 3 flags: precursor	3.38	0.010		
g12721	smc2	structural maintenance of chromosomes protein 2	3.32	0.000		
g12271	cspg2	versican core protein	3.12	0.000		
g12322	impg2	interphotoreceptor matrix proteoglycan 2	0.20	0.001		
g23617	chia	acidic mammalian chitinase	6.21	0.000	16.49	0.000
g34568	muc5a	mucin-5ac	3.02	0.002	24.74	0.000
g24192	muc5b	mucin-5b			41.31	0.000
g35363	muc5a	mucin-5ac			21.34	0.001
g28800	muc5b	mucin-5b			19.61	0.000
g18964	muc5a	mucin-5ac c			13.31	0.000
g19822	co5a1	collagen alpha-1 chain flags: precursor			0.33	0.005
g17364	tecta	alpha-tectorin flags: precursor			0.04	0.004

Fold cha. = Fold change; pval = 0.000 indicates P values < 0.0005

#### Angiogenesis or vasculogenesis

In infected yellow eels, 51 genes related to angiogenesis or vasculogenesis were modified, and only 9 of these genes were reduced in their expression level (Table 6). Expression of angiopoietin-related protein 7 (angl7), was switched on in infected silver eels, and connective tissue growth factor (ctgf), signal cub and egf-like domain containing protein (scub3), bone morphogenetic protein1 (bmp1), and several copies of thrombospondin (tsp1; tsp4b) were expressed at a significantly higher level.

**Table 6:** Differentially transcribed genes (fold change >3) based on GO terms “angiogenesis” or “vasculogenesis” in infected yellow and infected silver eels as compared with uninfected yellow and uninfected silver eels, respectively.

Gene	Name	Description	Yellow		Silver	
			Fold cha.	pval	Fold cha.	pval
g38341	angl7	angiopoietin-related protein 7	Inf	0.004		
g6637	cy24b	cytochrome b-245 heavy chain	9.01	0.001		
g10399	plcz1	1-phosphatidylinositol- -bisphosphate phosphodiesterase zeta-1	8.85	0.003		
g17733	fhr2	complement factor h-related protein 2	7.64	0.000		
g1691	tsp1	thrombospondin-1 flags: precursor	6.46	0.000		
g8980	agtr2	type-2 angiotensin ii receptor	5.26	0.001		
g3764	ctgf	connective tissue growth factor	5.14	0.000		
g7750	tsp4b	thrombospondin-4-b	4.97	0.000		
g1692	tsp1	thrombospondin-1 flags: precursor	4.84	0.000		
g17339	cxcr4	c-x-c chemokine receptor type 4	4.66	0.000		
g42042	tec	tyrosine-protein kinase tec	4.44	0.006		
g23394	frem2	fras1-related extracellular matrix protein 2	4.21	0.000		
g11400	junb	transcription factor jun-b	4.20	0.000		
g20805	co7	complement component c7 flags: precursor	4.11	0.000		
g3425	plcb2	1-phosphatidylinositol- -bisphosphate phosphodiesterase beta-2	4.08	0.002		
g12361	junb	transcription factor jun-b	4.06	0.000		
g7816	hmox	heme oxygenase	4.05	0.000		
g4725	cyr61	protein cyr61	4.00	0.000		
g16847	cytf	cystatin-f	3.98	0.000		
g11223	lyve1	lymphatic vessel endothelial hyaluronic acid receptor 1	3.97	0.000		
g21342	myo1f	myosin-if	3.96	0.000		
g513	sem3c	semaphorin-3c	3.96	0.001		
g21035	sem4b	semaphorin-4b flags: precursor	3.81	0.000		
g27449	c3p1	protein c3p1	3.80	0.000		
g20848	myo1f	myosin-if	3.73	0.000		
g29877	myo1f	myosin-if	3.68	0.004		
g8377	angl7	angiopoietin-related protein 7	3.65	0.001		
g35688	itb2	integrin beta-2	3.63	0.000		
g12898	ptprh	receptor-type tyrosine-protein phosphatase h	3.47	0.000		
g5296	cn073	sec6-like protein c14orf73	3.44	0.000		
g5094	scub3	signal cub and egf-like domain-containing protein 3 flags: precursor	3.38	0.010		
g18110	par11	poly polymerase 11	3.36	0.002		
g9669	bmp1	bone morphogenetic protein 1	3.34	0.000		
g21341	myo1e	myosin-ie	3.14	0.000		
g20847	myo1f	myosin-if	3.12	0.000		
g14921	ets1a	protein c-ets-1-a	3.11	0.003		
g7735	cy24b	cytochrome b-245 heavy chain	3.07	0.000		
g26070	co4	complement c4 contains:	3.06	0.000		
g1347	sh2d7	sh2 domain-containing protein 7	3.05	0.010		
g11499	ccl20	c-c motif chemokine 20	3.05	0.000		
g22834	f13a	coagulation factor xiii a chain	0.33	0.000		
g21936	npas4	neuronal pas domain-containing protein 4	0.29	0.003		
g3471	tnni2	troponin fast skeletal muscle	0.13	0.000		
g13604	cramp	cathelin-related antimicrobial peptide	0.13	0.000		
g6339	dlld	delta-like protein d	0.12	0.000		
g21922	s12a5	solute carrier family 12 member 5	0.10	0.004		
g13534	cxd2	gap junction delta-2 protein	0.08	0.000		
g16284	dscam	down syndrome cell adhesion molecule homolog flags: precursor	0.05	0.000		
g9077	tbxt	t-box-containing protein tbxt	0.04	0.000		
g22125	twhh	tiggy-winkle hedgehog protein	Inf	0.000	Inf	0.000
g12811	co3	complement c3 contains:	9.09	0.000	9.61	0.002
g10752	plxa4	plexin-a4 flags: precursor			Inf	0.003
g45052	pe2r1	prostaglandin e2 receptor ep1 subtype			25.69	0.004
g29481	mk11	mitogen-activated protein kinase 11			23.92	0.000
g41010	s1pr3	sphingosine 1-phosphate receptor 3			13.74	0.001
g26552	robo2	roundabout homolog 2 flags: precursor			12.78	0.001
g36054	s1pr4	sphingosine 1-phosphate receptor 4			12.46	0.000
g44943	mk11	mitogen-activated protein kinase 11			9.97	0.002
g6564	prg4	proteoglycan 4			9.65	0.000
g20875	pf2r	prostaglandin f2-alpha receptor			6.82	0.009
g39633	co5	complement c5			6.32	0.008
g15048	co3	complement c3 contains:			6.13	0.003
g15757	ampe	glutamyl aminopeptidase			6.03	0.004
g18890	ampn	aminopeptidase n			5.06	0.010
g19822	co5a1	collagen alpha-1 chain flags: precursor			0.33	0.005
g35655	cxl10	c-x-c motif chemokine 10			0.23	0.003
g8995	scub2	signal cub and egf-like domain-containing protein 2			0.20	0.004
g8625	hxd3a	homeobox protein hox-d3a			0.12	0.008
g24694	mmp17	matrix metalloproteinase-17			0.08	0.000

Fold cha. = Fold change; pval = 0.000 indicates P values < 0.0005

#### Immune defense

The largest number of genes affected by the infection of the swimbladder with the nematode was related to immune defense. In infected yellow eels, 167 genes were modified in their expression level, and only 24 of these genes were reduced in their expression level (Table 7). Many-fold elevated in their expression level were genes coding for immunoglobulin light chain, immunoglobulin heavy chain variable region, complement proteins (co3; cfah; fhr2; c1r; co4a; co7), several interleukins (interleukin 12subunit beta, il12b; interleukin-18 receptor 1, il18r; interleukin-6 receptor subunit beta, il6rb; interleukin-17 receptor b, i17rb), and interferon regulatory factor (irf4). In addition, several heat shock proteins showed increased mRNA expression levels (heat shock 70 kda, hsp70; heat shock protein beta, hspbb; heat shock protein 105 kda, hs105).

**Table 7:** Differentially transcribed genes (fold change >3) based on GO terms related to “immune defense” in infected yellow and infected silver eels as compared with uninfected yellow and uninfected silver eels, respectively.

Gene	Name	Description	Yellow		Silver	
			Fold cha.	pval	Fold cha.	pval
g38341	angl7	angiopoietin-related protein 7	Inf	0.004		
g13085	mlrv	myosin regulatory light chain ventricular cardiac muscle isoform	26.82	0.005		
g11898	fosb	protein fosb	21.55	0.000		
g16766	i23o1	indoleamine -dioxygenase 1	18.77	0.001		
g43990	ns1ba	influenza virus ns1a-binding protein homolog a	16.15	0.002		
g14949	co3	complement c3 contains:	12.91	0.000		
g38820	gima1	gtpase imap family member 1	12.32	0.008		
g41857		immunoglobulin heavy chain variable region	12.27	0.000		
g31061		immunoglobulin light chain	12.00	0.008		
g34285	lv302	ig lambda chain v-iii region loi	10.25	0.000		
g37595	hsp70	heat shock 70 kda protein	10.04	0.000		
g5407	hspbb	heat shock protein beta-11	10.00	0.001		
g6637	cy24b	cytochrome b-245 heavy chain	9.01	0.000		
g34987	fucl4	fucolectin-4 flags: precursor	8.42	0.000		
g30502	gima4	gtpase imap family member 4	8.37	0.000		
g2443	rgs4	regulator of g-protein signaling 4	8.15	0.000		
g34201		mhc class i antigen	7.87	0.000		
g22923	cfah	complement factor h	7.80	0.000		
g17733	fhr2	complement factor h-related protein 2	7.64	0.000		
g9234	c1qrf	c1q-related factor	7.61	0.010		
g10835	hs105	heat shock protein 105 kda	7.57	0.000		
g42422	igkc	ig kappa chain c region	7.56	0.000		
g24709	vsig1	v-set and immunoglobulin domain-containing protein 1	7.55	0.005		
g20323	il12b	interleukin-12 subunit beta	7.21	0.000		
g35498		immunoglobulin light chain	6.86	0.000		
g9306	dnjb4	homolog subfamily b member 4	6.64	0.000		
g1691	tsp1	thrombospondin-1 flags: precursor	6.46	0.000		
g16469	tri69	tripartite motif-containing protein 69	6.12	0.000		
g26875	lysc	lysozyme c	5.86	0.000		
g10077	c1r	complement c1r subcomponent	5.62	0.000		
g16623	tutlb	protein turtle homolog b	5.62	0.004		
g20076	cd3g	t-cell surface glycoprotein cd3 gamma chain	5.58	0.000		
g6900	nfac2	nuclear factor of activated t- cytoplasmic 2	5.46	0.000		
g39678	irf4	interferon regulatory factor 4	5.38	0.000		
g24053	gima4	gtpase imap family member 4	5.21	0.000		
g36156	ha1d	h-2 class i histocompatibility k-d alpha chain	5.16	0.000		
g11916	tcc4	t-cell receptor gamma chain c region 5 10–13	5.09	0.000		
g12086	l3bpb	galectin-3-binding protein b	5.06	0.000		
g22618	chia	acidic mammalian chitinase	5.05	0.000		
g24469	elf3	ets-related transcription factor elf-3	5.02	0.001		
g42742		mhc class i antigen	5.00	0.000		
g1342	dnja4	homolog subfamily a member 4	4.99	0.000		
g7750	tsp4b	thrombospondin-4-b	4.97	0.000		
g23321	ccr9	c-c chemokine receptor type 9	4.97	0.000		
g12409	fos	proto-oncogene c-fos	4.96	0.000		
g26051	zap70	tyrosine-protein kinase zap-70	4.91	0.000		
g5410	hspbb	heat shock protein beta-11	4.84	0.000		
g1493	rgs8	regulator of g-protein signaling 8	4.83	0.000		
g23965	lac6	ig lambda-6 chain c region	4.72	0.000		
g10121	lck	tyrosine-protein kinase lck	4.67	0.000		
g1848	ccl25	c-c motif chemokine 25	4.66	0.000		
g17339	cxcr4	c-x-c chemokine receptor type 4	4.66	0.000		
g40079		immunoglobulin light chain	4.66	0.000		
g278	tnfa	tumor necrosis factor	4.64	0.004		
g32441	smp	schwann cell myelin protein	4.62	0.000		
g10172	slap2	src-like-adapter 2	4.59	0.000		
g19466	irf4	interferon regulatory factor 4	4.54	0.000		
g23648	sepr	seprase	4.52	0.000		
g45177	l3bpb	galectin-3-binding protein b	4.52	0.000		
g21248	ccl4	c-c motif chemokine 4	4.44	0.000		
g14635	gima7	gtpase imap family member 7	4.44	0.000		
g42042	tec	tyrosine-protein kinase tec	4.44	0.006		
g12085	l3bpb	galectin-3-binding protein b	4.43	0.000		
g34980	lysc3	lysozyme c-3	4.37	0.000		
g8265	frim	middle subunit short = ferritin m	4.29	0.000		
g41703	zap70	tyrosine-protein kinase zap-70	4.23	0.001		
g23394	frem2	fras1-related extracellular matrix protein 2	4.21	0.000		
g1769	il2rg	cytokine receptor common subunit gamma	4.20	0.000		
g40915	gima4	gtpase imap family member 4	4.12	0.000		
g20805	co7	complement component c7 flags: precursor	4.11	0.000		
g22235	ibp3	insulin-like growth factor-binding protein 3	4.06	0.000		
g7816	hmox	heme oxygenase	4.05	0.000		
g1450	cd22	b-cell receptor cd22	4.04	0.000		
g2603	ten4	teneurin-4	3.99	0.009		
g9327	il18r	interleukin-18 receptor 1	3.99	0.006		
g16847	cytf	cystatin-f	3.98	0.000		
g11223	lyve1	lymphatic vessel endothelial hyaluronic acid receptor 1	3.97	0.000		
g21342	myo1f	myosin-if	3.96	0.000		
g18814	ylat2	y+l amino acid transporter 2	3.91	0.000		
g25574	tnr9	tumor necrosis factor receptor superfamily member 9	3.87	0.000		
g2215	chst1	carbohydrate sulfotransferase 1	3.87	0.000		
g17581	lox5	arachidonate 5-lipoxygenase	3.86	0.001		
g2197	bc11b	b-cell lymphoma leukemia 11b	3.86	0.000		
g3335	p2y14	p2y purinoceptor 14	3.81	0.003		
g21035	sem4b	semaphorin-4b flags: precursor	3.81	0.000		
g27449	c3p1	protein c3p1	3.80	0.000		
g3210	syub	beta-synuclein	3.77	0.005		
g16262	ciks	adapter protein ciks	3.76	0.000		
g44240	hvm45	ig heavy chain v region mc101 flags: precursor	3.75	0.000		
g20848	myo1f	myosin-if	3.73	0.000		
g23368	il6rb	interleukin-6 receptor subunit beta	3.71	0.000		
g39609	igkc	ig kappa chain c region	3.70	0.000		
g3322	fos	proto-oncogene c-fos	3.70	0.000		
g29877	myo1f	myosin-if	3.68	0.004		
g14600	gpr4	g-protein coupled receptor 4	3.65	0.000		
g36379	co4a	complement c4-a	3.64	0.000		
g35688	itb2	integrin beta-2	3.63	0.000		
g30636	ajl1	galactose-binding lectin l-1	3.62	0.000		
g11126	lr16b	leucine-rich repeat-containing protein 16b	3.61	0.000		
g38019	ksyk	tyrosine-protein kinase syk	3.59	0.000		
g4585	a3lt2	alpha- -galactosyltransferase 2	3.57	0.002		
g26488	nckpl	nck-associated protein 1-like	3.49	0.000		
g12898	ptprh	receptor-type tyrosine-protein phosphatase h	3.47	0.000		
g27889	fyn	tyrosine-protein kinase fyn	3.46	0.000		
g26288	nckpl	nck-associated protein 1-like	3.45	0.000		
g8551	nfkb1	nuclear factor nf-kappa-b p105 subunit	3.44	0.001		
g36600	cml1	chemokine-like receptor 1	3.43	0.000		
g41695	hmr1	major histocompatibility complex class i-related gene protein	3.43	0.000		
g12291	rac2	ras-related c3 botulinum toxin substrate 2	3.42	0.000		
g28849	igg2b	ig gamma-2b chain c region	3.41	0.000		
g5094	scub3	signal cub and egf-like domain-containing protein 3 flags: precursor	3.38	0.010		
g39226	gima4	gtpase imap family member 4	3.38	0.000		
g18110	par11	poly polymerase 11	3.36	0.002		
g9299	gp183	g-protein coupled receptor 183	3.36	0.000		
g21657	perf	perforin-1	3.34	0.000		
g12497	kpcb	protein kinase c beta type	3.34	0.004		
g16269	svep1	von willebrand factor type egf and pentraxin domain-containing protein 1	3.33	0.006		
g1218	dock2	dedicator of cytokinesis protein 2	3.30	0.000		
g264	aif1l	allograft inflammatory factor 1-like	3.29	0.000		
g35485	gima7	gtpase imap family member 7	3.29	0.000		
g23133	pi2r	prostacyclin receptor	3.28	0.000		
g13206	fbx40	f-box only protein 40	3.26	0.000		
g25649	tutla	protein turtle homolog a	3.25	0.002		
g5757	plcg2	1-phosphatidylinositol- -bisphosphate phosphodiesterase gamma-2	3.23	0.000		
g15532	cml1	chemokine-like receptor 1	3.22	0.000		
g11604	ccl19	c-c motif chemokine 19	3.21	0.000		
g4039	cdk1	cell division protein kinase 1	3.19	0.000		
g43465	co7	complement component c7 flags: precursor	3.17	0.000		
g21341	myo1e	myosin-ie	3.14	0.000		
g41479	nalp1	lrr and pyd domains-containing protein 1	3.14	0.000		
g20847	myo1f	myosin-if	3.12	0.000		
g14921	ets1a	protein c-ets-1-a	3.11	0.003		
g4833	il6ra	interleukin-6 receptor subunit alpha	3.10	0.000		
g7735	cy24b	cytochrome b-245 heavy chain	3.07	0.000		
g26070	co4	complement c4 contains:	3.06	0.000		
g11499	ccl20	c-c motif chemokine 20	3.05	0.000		
g12107	grn	granulins	3.05	0.000		
g9518	tec	tyrosine-protein kinase tec	3.04	0.000		
g2101	thms1	protein themis	3.01	0.000		
g22834	f13a	coagulation factor xiii a chain short = coagulation factor xiiia	0.33	0.000		
g16693	pamr1	inactive serine protease pamr1	0.31	0.000		
g23774	ppara	peroxisome proliferator-activated receptor alpha short = ppar-alpha	0.31	0.005		
g3029	tri25	e3 ubiquitin isg15 ligase trim25	0.29	0.000		
g21936	npas4	neuronal pas domain-containing protein 4	0.29	0.003		
g13774	ap1s2	ap-1 complex subunit sigma-2	0.28	0.002		
g28238	pvrl3	poliovirus receptor-related protein 3-like flags: precursor	0.26	0.000		
g15087	actc	alpha cardiac muscle 1	0.25	0.005		
g10993	s100p	protein s100-p	0.23	0.000		
g19703	cadm3	cell adhesion molecule 3	0.19	0.005		
g6116	acts	alpha skeletal muscle	0.18	0.000		
g30946	gima7	gtpase imap family member 7	0.17	0.000		
g21654	h1	histone h1 contains:	0.17	0.000		
g16176	pdyn	proenkephalin-b	0.16	0.000		
g28779	cadm3	cell adhesion molecule 3	0.16	0.002		
g31407	gima4	gtpase imap family member 4	0.16	0.000		
g2246	mk10	mitogen-activated protein kinase 10	0.14	0.000		
g21392	nalp1	lrr and pyd domains-containing protein 1	0.13	0.000		
g13604	cramp	cathelin-related antimicrobial peptide	0.13	0.000		
g2030	pa24c	cytosolic phospholipase a2 gamma	0.12	0.000		
g3113	hxk4	glucokinase	0.08	0.009		
g11642	cof2	cofilin-2	0.06	0.000		
g25966	gima5	gtpase imap family member 5	0.05	0.003		
g32493		mhc class i antigen	0.04	0.000		
g9182	scn5a	sodium channel protein type 5 subunit alpha	0.03	0.000		
g11847	lyg	lysozyme g	0.01	0.000		
g22125	twhh	tiggy-winkle hedgehog protein	Inf	0.000	Inf	0.000
g2358	noxo1	nadph oxidase organizer 1	11.27	0.000	25.87	0.000
g14663	cp1b1	cytochrome p450 1b1	11.08	0.000	0.22	0.002
g12811	co3	complement c3 contains:	9.09	0.000	9.61	0.002
g23617	chia	acidic mammalian chitinase	6.21	0.000	16.49	0.000
g7584	cxcr1	c-x-c chemokine receptor type 1	5.74	0.000	23.70	0.000
g14950	co3	complement c3 contains:	5.44	0.000	4.68	0.000
g20618	il6rb	interleukin-6 receptor subunit beta	4.78	0.000	6.26	0.001
g605	i17rb	interleukin-17 receptor b	4.17	0.000	7.52	0.001
g3715	lpar6	lysophosphatidic acid receptor 6	4.02	0.002	6.88	0.000
g513	sem3c	semaphorin-3c	3.96	0.001	41.77	0.002
g24625	clm3	cmrf35-like molecule 3	3.92	0.000	9.47	0.001
g24330	dclk2	serine threonine-protein kinase dclk2	3.56	0.002	8.15	0.000
g34568	muc5a	mucin-5ac	3.02	0.002	24.74	0.000
g16142	ticn1	testican-1	0.21	0.000	16.89	0.000
g23142	hecw1	e3 ubiquitin-protein ligase hecw1			Inf	0.002
g16672	smoc1	sparc-related modular calcium-binding protein 1			Inf	0.003
g26738	hfe	hereditary hemochromatosis protein			24.64	0.000
g29481	mk11	mitogen-activated protein kinase 11			23.92	0.000
g35363	muc5a	mucin-5ac short = muc-5ac			21.34	0.001
g34977	ffar2	free fatty acid receptor 2			20.05	0.000
g25084	pnph	purine nucleoside phosphorylase			17.45	0.001
g30308	dclk2	serine threonine-protein kinase dclk2			16.53	0.001
g11737	noxo1	nadph oxidase organizer 1			15.96	0.000
g22925	dclk2	serine threonine-protein kinase dclk2			15.11	0.001
g45517	argn3	non-hepatic 3			15.02	0.001
g41010	s1pr3	sphingosine 1-phosphate receptor 3			13.74	0.001
g36054	s1pr4	sphingosine 1-phosphate receptor 4			12.46	0.000
g26998	bpi	bactericidal permeability-increasing protein			10.12	0.000
g44943	mk11	mitogen-activated protein kinase 11			9.97	0.002
g6564	prg4	proteoglycan 4			9.65	0.000
g838	ptx3	pentraxin-related protein ptx3			9.24	0.000
g22135	tlr1	toll-like receptor 1			8.25	0.004
g28036	gima5	gtpase imap family member 5			8.13	0.001
g28682	siat2	beta-galactoside alpha- -sialyltransferase 2			7.50	0.010
g39633	co5	complement c5			6.32	0.008
g15048	co3	complement c3 contains:			6.13	0.003
g27549	ita2	integrin alpha-2			5.92	0.004
g5914	dmbt1	deleted in malignant brain tumors 1 protein			5.17	0.002
g11202	crld2	cysteine-rich secretory protein lccl domain-containing 2 flags: precursor			5.11	0.004
g14464	aqp3	aquaporin-3			4.44	0.002
g20649	argi2	arginase- mitochondrial			3.88	0.007
g23306	gima4	gtpase imap family member 4			3.78	0.007
g21455	pnph	purine nucleoside phosphorylase			3.72	0.001
g30996		mhc class i antigen			0.28	0.004
g9834	cadm1	cell adhesion molecule 1			0.26	0.002
g35655	cxl10	c-x-c motif chemokine 10			0.23	0.003
g16242	cfab	complement factor b			0.22	0.001
g1090	ileu	leukocyte elastase inhibitor			0.22	0.003
g8995	scub2	signal cub and egf-like domain-containing protein 2			0.20	0.004
g18096	ubc4	probable bifunctional e2 e3 enzyme r795 includes:			0.18	0.008
g26753	cxl11	c-x-c motif chemokine 11			0.17	0.000
g14172	cadm2	cell adhesion molecule 2			0.16	0.004
g10119	mbl2	mannose-binding protein c			0.16	0.000
g36679	galt8	probable polypeptide n-acetylgalactosaminyltransferase 8			0.15	0.001
g7564	uchl1	ubiquitin carboxyl-terminal hydrolase isozyme l1			0.14	0.009
g25543	gima7	gtpase imap family member 7			0.10	0.000
g37466	hmr1	major histocompatibility complex class i-related gene protein			0.10	0.000
g4910	s100b	protein s100-b			0.09	0.005
g24694	mmp17	matrix metalloproteinase-17			0.08	0.000
g31085	gbp5	guanylate-binding protein 5			0.05	0.000
g17364	tecta	alpha-tectorin flags: precursor			0.04	0.004
g31792	lv302	ig lambda chain v-iii region loi			0.02	0.000
g1076	cats	cathepsin s flags: precursor			0.01	0.000
g1271	h2b3	Histone			0.00	0.002

Fold cha. = Fold change; pval = 0.000 indicates P values < 0.0005

#### Maturation

Of the genes related to maturation, 67 were modified in their expression level, and 15 of these genes showed reduced expression levels (S2 Table). Two copies of fer-1-like protein 4 (fr1l4) were 16- and 25-fold elevated in the mRNA expression level. A number of genes listed under the GO term maturation has also been listed under different GO terms, like, for example angl7; protein fsb, fos, hspbb, tsp1, tsp4b, gtr5, cftr.

### Transcriptional changes in silver eel gas gland tissue related to the nematode infection

#### Glucose metabolism

Overall, 10 genes of glucose and lactate metabolism were affected by the infection in yellow eels, while only 4 genes were affected in silver eels (Table 3). None of the genes involved in glycolysis was affected in infected silver eels, and only one glucose transport and one monocarboxylate transporter showed a higher mRNA expression level.

#### ROS defense

While 40 genes related to ROS were affected in the mRNA expression level in infected yellow eels, only 6 genes were affected in infected silver eels (Table 4). Among these 6 genes matrix metalloproteinase-9 (mmp9) and hereditary hemochromatosis protein (hfe) showed a more than 20-fold increased expression level in infected silver eels, while the other 4 genes showed largely reduced expression levels. Cytochrome p450 1b1 (cp1b1), which was significantly elevated in infected yellow eels, was about 5-fold downregulated in infected silver eels.

#### Ion transport

In infected silver eels gas gland tissue, 19 genes showed modified expression levels, with 5 downregulated and 14 upregulated genes (S1 Table). Only 2 ion transport proteins were modified in the expression level in infected silver eels gas gland cells, and, as already observed in infected yellow eels, the expression level of sodium potassium-transporting atpase was largely reduced, but in contrast to yellow eels, in silver eels subunit gamma (atng) was affected. In infected silver eels, the amino acid transporters showed increased mRNA expression levels (sodium and chloride-dependent neutral and basic amino acid transporter b(0+), s6a14; excitatory amino acid transporter 2, eaa2).

#### Extracellular matrix

In infected silver eels, 8 genes related to the extracellular matrix were modified, but only two of these genes (acidic mammalian chitinase, chia, and mucin 5b, muc5b) were also affected in infected yellow eels (Table 5). In contrast to infected yellow eels, 4 additional mucin genes showed an increased expression level. In fact, in silver eels 5 out of 8 affected genes were mucin genes. Collagen alpha-1 (co5a1) was expressed at a 3-fold lower level in infected silver eel gas gland cells.

#### Angiogenesis or vasculogenesis

In infected silver eels, the number of genes modified with respect to angiogenesis or vasculogenesis was much smaller than in infected yellow eels (20 and 51 genes, respectively) (Table 6), and of these genes only tiggy-winkle hedgehog protein (twhh) and complement c3 (co3) were affected in yellow as well as in silver eels. Expression of prostaglandine2 receptor (pe2r1), of sphingosine receptors (s1pr3; s1pr4), and of roundabout homolog 2 (robo2) was elevated, and mRNA of complement proteins was increased (co3, co5). The expression level of angiopoietin was not affected by the nematode infection.

#### Immune defense

Compared to infected yellow eels, the immune related changes were much less pronounced in infected silver eels (Table 7). In infected silver eels only 64 genes were expressed at a different level, and 21 of these genes were downregulated. Only two of the interleukin genes were elevated in their expression level (il6rb, i17rb), and immunoglobulin genes were unaffected. As observed in infected yellow eels, two genes coding for complement proteins (co3; co5) were elevated in their expression level, but complement factor b (cfab) was more than 4-fold reduced in the expression level. Major histocompatibility complex class I related gene (hmr1) was even 10-fold decreased in the expression level.

#### Maturation

Of the genes related to maturation, 25 genes were modified in their expression level in infected silver eels, and 13 of these genes decreased (S2 Table). As observed in infected yellow eels, two copies of fer-1-like protein 4 (fr1l4) were elevated in their expression level (11-fold and 37-fold). The expression of three zona pellucida genes (zp1, zp2, zp3) was more than 100-fold reduced.

Table 8 summarizes the number of genes related to specific physiological functions expected to be important for swimbladder function and modified in their expression level in infected yellow and silver eels. The comparison clearly showed that in infected yellow eels, many more genes were affected, compared to infected silver eels. Furthermore, the number of genes affected in both, infected yellow and silver eels, was very small, indicating that, depending on the developmental stage, different sets of genes were affected.

**Table 8:** Overview of the pathways analyzed (Tables 3–7 and S1 and S2 Tables) and the total number of genes affected in infected yellow eels and in infected silver eels.

GO term	Inf. yellow	Inf. silver	Common	Infected yellow		Infected silver	
				Up	Down	Up	Down
Glucose metabolism	10	4	1	7	3	3	1
ROS metabolism	40	6	3	32	8	4	2
Extracellular matrix	11	8	2	10	1	6	2
Ion exchange	56	19	6	38	18	13	6
Angiogenesis	51	20	2	42	9	15	5
Immune response	167	64	15	143	24	43	21
Maturation	67	25	6	52	15	12	13

The table also shows the number of genes affected in both, infected yellow and in infected silver eel gas gland tissue (common), and the number of up and downregulated genes in both groups.

## Discussion

### Transcriptional changes observed in infected yellow eel gas gland tissue

In a previous study we addressed the transcriptional changes related to silvering in uninfected European eels, and at a significance level of P < 0.01, 646 genes were found to be transcribed at a different level [26]. The present study showed that the influence of an infection of the yellow eel swimbladder with the nematode *Anguillicola crassus* on transcriptional activity in gas gland cells by far exceeded the effect of silvering. In infected yellow eel gas gland tissue, 1675 genes were modified in their mRNA expression level. As expected, GO enrichment analysis revealed that the most prominent category was immune response with 143 genes expressed at a higher level and only 24 genes expressed at a lower level. The large fraction of genes with elevated expression level included various inflammatory components, complement proteins, and immunoglobulins, indicating a strong defense reaction of the eel. An extensive non-specific immune response has been reported in response to juvenile nematodes/parasites entering the swimbladder [50], and Nimeth et al. [51] demonstrated that even glass eels can be infected by feeding on copepods. An activation of the immune system in infected eels has previously been suggested by presence of macrophages in swimbladder tissue [52–54]. Experimental infections of the swimbladder have also been reported to cause a humoral response [55]. An infection of the swimbladder with the histophagous nematode results in severe histological modifications of the swimbladder epithelium [27,29–31,56]. The single layered epithelium of the eel becomes severely thickened and multilayered. Signs of tissue degeneration appear, and the lumen is filled with eggs, larvae, and exudate. Ultimately, these effects can lead to a total loss of swimbladder function [29]. The elevated expression of acidic mammalian chitinase among the extracellular matrix components also can be interpreted as an immune response to the nematode infection. Chitin is a surface component of parasites and induces the expression of chitinase in the host [57]. MMP9 expression is also elevated in infected eels, and this protein has been shown to be an essential component of the innate immune system [58].

More recent observations suggest that the infection rate may stabilize [59], and eels with thickened swimbladder wall, but with very few or even no nematode inside the bladder indicate that the mechanical barrier, combined with the inflammatory response, may be successful in defending the nematode [37].

Thickening of the tissue in response to the infection results in larger diffusion distances. The elevated expression levels of glucose transporters and of monocarboxylate transport proteins, and in particular of fructose-bisphosphate aldolase suggested a stimulation of glycolytic activity. Fructose-bisphosphate aldolase is known as a key enzyme for glycolytic flux. Glucokinase, in turn, was found with largely reduced copy numbers in infected yellow eel swimbladder. In swimbladder tissue of cod, hexokinase appears to be the key enzyme for phosphorylation of glucose taken up from the blood [60]. Therefore, the reduced expression rate of glucokinase, an enzyme of crucial importance in liver tissue, may not compromise glycolytic flux in gas gland tissue.

The elevated expression level of a number of genes related to the extracellular matrix, including collagen alpha, versican, and two thrombospondins, appeared to be connected to the thickening of the swimbladder tissue. Collagen is a typical component of the extracellular matrix. The proteoglycan versican has been reported to be expressed by vascular smooth muscle cells [61], and the glycoprotein thrombospondin has been shown to inhibit angiogenesis and neovascularization [62]. The thickening of the gas gland epithelium obviously coincided with an increase in extracellular matrix in infected eels.

The induction of Angiopoietin-related protein in infected eels also appeared to be connected to tissue thickening. In contrast to thrombospondin, which inhibits angiogenesis, angiopoietin-related protein 7 has been shown to induce sprouting in endothelial cells [63], which would reduce diffusion distances and therefore improve nutrient and oxygen supply to the tissue.

Ion regulation and in particular acid secretion is crucial for swimbladder functioning [64–66], and in infected yellow eels a number of ion transporters were modified in their expression level. Several Na^+^, K^+^, and Cl^-^ transport proteins were affected, but the expression changes were not consistent. While 7 mRNA species showed elevated levels, 6 were significantly reduced. V-ATPase and Na^+^/H^+^ exchange proteins were not affected, suggesting that acid secretion in particular was not seriously modified [64,65]. Interestingly, sodium-potassium atpase subunit beta-2 was more than 8-fold reduced in the expression level. As many ion transport processes require Na^+^/K^+^-ATPase activity as a second step, this suggested that overall ion transport activity was not enhanced by the infection.

ROS and ROS defense play a special role in swimbladder tissue due to the high oxygen partial pressures encountered [32], and several genes related to the GO term ROS defense were affected in their expression level. Genes particularly important for the degradation of ROS like glutathione reductase, glutathione peroxidase and superoxide dismutase were not among the modified genes, but a number of transcription factors like fos, fosb, and junb were affected by the infection. These transcription factors may be involved in a number of different physiological functions and signaling cascades, so that this result may not be indicative of a special enhancement of ROS defense in infected yellow eels. Jun and Fos family members heterodimerize to form Activator Protein 1 (AP1), which has a major role in tissue regeneration. Some of the observed expression changes may thus be secondary effects due to the formation of the AP1 complex [67–69]. The elevated expression levels of two cytochromes may, however, again reveal a connection to a defense reaction of the host, since cytochrome b245 has been connected to superoxide production and phagocyte activity [70], and cytochrome p450 is involved in detoxification [71]. Accordingly, the elevated expression levels of these enzymes again provide a strong indication for the defense reaction of the host against the infection.

As already observed in a previous study focusing on the effect of silvering on transcriptional activity [26], an infection with the nematode caused modifications in the expression level of genes related to maturation in swimbladder tissue. Several of these proteins were also listed under different GO terms, like transporters (gtr5, cftr) and a transcription factor (fos), so that a specific connection to maturation may not be obligatory in this tissue. Noteworthy was the elevation of fer-like proteins, which have previously been connected to vesicle fusion and membrane trafficking [72]. Ferlins represent an ancient protein family and appear to be of general importance for these membrane processes.

### Transcriptional changes observed in infected silver eel gas gland tissue

An initial comparison of the transcriptional effects observed in infected yellow eels with the effects detected in infected silver revealed large scale differences: while 1675 genes were differentially expressed in infected yellow eels, only 291 genes were affected in infected silver eels. Only a third of the genes modified in silver eels was also affected in yellow eels. Twenty-two of these genes, however, showed the opposite response in yellow compared with silver eels, supporting the impression that the nematode infection provoked quite different responses in yellow and silver eels.

Expressed at elevated levels in yellow eels but reduced in silver eels were zinc binding proteins. Zinc metalloenzyms are, for example, carboanhydrase, superoxide dismutase, collagenase, and elastase, enzymes that are important for the acidification of blood during passage of the swimbladder, for ROS defense and reconstruction of the extracellular matrix [18,65]. The elevated expression level of these enzymes in yellow eels would support swimbladder function, and thus could indicate that, in addition to the strong immune defense reaction, yellow eels attempted to retain a functional swimbladder. In infected silver eels, in turn, Ca^2+^ binding enzymes showed elevated expression levels. Ca^2+^ is a pivotal signaling component [73], but with respect to swimbladder function the role of Ca^2+^ does not appear to be crucial.

The conclusion that in infected silver eels transcriptional changes were not supportive for swimbladder function was underlined by the observation that in contrast to infected yellow eels, in infected silver eels, genes involved in glycolysis were not affected, and in addition, there was almost no response in genes involved in ROS defense. Both, glycolysis and ROS defense, however, are crucial for swimbladder function [18,19,32].

In infected silver eel gas gland tissue, the compared to infected yellow eels reduced responses of inflammatory components, of complement proteins and the reduced expression level of major histocompatibility complex revealed a very much reduced immune defense reaction. Silvering requires severe physiological reorganization, not only in gas gland cells [26], but also in terms of ion regulation to prepare for the transition to the marine environment. In addition, maturation is prepared [38]. These modifications require a lot of energy, which could result in reduced capacities for the immune response.

In line with these considerations, only few genes related to the GO term ‘ion regulation’ were differentially expressed in infected silver eels. Only two genes related to Na^+^, K^+^, and Cl^-^ transport were modified, and a subunit of Na^+^/K^+^-ATPase was reduced in the mRNA expression level, indicating that ion transport activity overall was reduced.

In a previous study we detected that at least in some uninfected silver eels, zona pellucida genes showed a significantly elevated expression level compared to uninfected yellow eels [26]. The present results revealed a significant reduced expression level in infected silver eels, as compared to uninfected one’s. These results supported the conclusion that silvering does include the onset of sexual maturation, and an elevation in plasma steroid concentrations [44] may have induced expression changes of maturation connected genes not only in gonads, but in other tissues as well.

The results of the present study revealed a very strong effect of the *Anguillicola crassus* infection on gas gland tissue of yellow eels, and compared to these changes in the mRNA expression the changes observed in infected silver eel gas gland tissue were very small, almost negligible. The largest difference in the response was observed in the immune response. In addition, some of the expression changes in infected yellow eels indicated an attempt to keep the swimbladder functional, but this was totally absent in infected silver eels. A possible explanation for this difference could be the silvering process. Silvering not only includes an improvement of swimbladder function [22–25,74], but also a total rearrangement of ion regulation to prepare for the switch to the marine environment, and the onset of maturation or puberty [38,41,75]. This could require so much energy and so many resources that there is not much scope to cope with the additional challenge of a nematode infection.

Another possible explanation is related to swimbladder function. The silvering event has been shown to improve swimbladder function [23], and this appears essential to prepare the swimbladder for the excessive changes in hydrostatic pressure, encountered during the vertical migrations taking place during the spawning migration [13,76]. On the other hand, theoretical considerations [19] demonstrated that it is impossible that the swimbladder can keep a constant volume throughout a six month journey to the Sargasso Sea (perhaps even longer; [14]) with daily vertical migrations between 200 or 300 m depth at night time, and 600–800 m depth at day time. Therefore, it is expected that the swimbladder provides neutral buoyancy near the upper level of the daily migrations, and is compressed during the descent to lower levels. If this is correct, the swimbladder volume must be adjusted to the upper level, and then the volume should be kept constant, which could be achieved by reducing gas loss through the swimbladder wall. Permeability of the swimbladder is in fact reduced during silvering [24], and this was supported by changes in the mRNA levels of genes related to the extracellular matrix in silver eels, as compared to yellow eels [26]. In this situation, gas-secreting activity of the bladder could be largely reduced, which could coincide with a downregulation of metabolic activity and a reduced responsiveness to other challenges, like a nematode infection.

## Supporting information

S1 Table Differentially transcribed genes (fold change >3) based on GO term “ion transport” in infected yellow and infected silver eels as compared with uninfected yellow and uninfected silver eels, respectively. (DOCX)

S2 Table Differentially transcribed genes (fold change >3) based on GO terms related to “maturation” in infected yellow and infected silver eels as compared with uninfected yellow and uninfected silver eels, respectively. (DOCX)

## References

[1] Rousseau K, Aroua S, Dufour S. Eel Secondary Metamorphosis: Silvering In: Dufour S, Rousseau K, Kapoor BG, editors. Metamorphosis in Fish. Boca Raton, FL: CRC Press, Taylor & Francis Group; 2012 pp. 216–249.

[2] Schmidt J. Breeding Places and Migrations of the Eel. Nature. 1923;111: 51–54. doi: 10.1038/111051a0

[3] Miller MJ, Bonhommeau S, Munk P, Castonguay M, Hanel R, McCleave JD. A century of research on the larval distributions of the Atlantic eels: a re-examination of the data. Biol Rev. 2015;90: 1035–1064. doi: 10.1111/brv.12144 25291986 10.1111/brv.12144

[4] Dekker W. What caused the decline of the Lake IJsselmeer eel stock after 1960? ICES J Mar Sci. 2004;61: 394–404. doi: 10.1016/j.icesjms.2004.01.003

[5] Kettle AJ, Vøllestad LA, Wibig J. Where once the eel and the elephant were together: decline of the European eel because of changing hydrology in southwest Europe and northwest Africa? Fish Fish. 2011;12: 380–411. doi: 10.1111/j.1467-2979.2010.00400.x

[6] Geeraerts C, Belpaire CGJ. The effects of contaminants in European eel: a review. Ecotoxicology. 2010;19: 239–266. doi: 10.1007/s10646-009-0424-0 19806452 10.1007/s10646-009-0424-0

[7] Baltazar-Soares M, Biastoch A, Harrod C, Hanel R, Marohn L, Prigge E, et al Recruitment collapse and population structure of the European eel shaped by local ocean current dynamics. Curr Biol. 2014;24: 104–108. doi: 10.1016/j.cub.2013.11.031 24374306 10.1016/j.cub.2013.11.031

[8] Bonhommeau S, Chassot E, Planque B, Rivot E, Knap AH, Le Pape O. Impact of climate on eel populations of the Northern Hemisphere. Mar Ecol Prog Ser. 2008;373: 71–80. doi: 10.3354/meps07696

[9] Lefebvre F, Fazio G, Crivelli AJ. *Anguillicoloides crassus* In: Woo P, Buchmann K, editors. Fish Parasites: Pathobiology and Protection. London: CAB International; 2012 pp. 310–326. http://www.uoguelph.ca/~pwoo/FPPP.description.pdf

[10] Bandín I, Souto S, Cutrín JM, López-Vázquez C, Olveira JG, Esteve C, et al Presence of viruses in wild eels *Anguilla anguilla L*, from the Albufera Lake (Spain). J Fish Dis. 2014;37: 597–607. doi: 10.1111/jfd.1392 24846700 10.1111/jfd.1392

[11] ICES 2016. Report of the Working Group on Eels (WGEEL), 15–22 September 2016, Cordoba, Spain. ICES CM 2016/ACOM:19. 107 pp.

[12] Jacoby D, Gollock M. *Anguilla anguilla*. IUCN Red List Threat Species 2014. The IUCN Red List of Threatened Species 2014; 2014;e.T60344A4.

[13] Aarestrup K, Okland F, Hansen MM, Righton DA, Gargan P, Castonguay M, et al Oceanic Spawning Migration of the European Eel (*Anguilla anguilla*). Science. 2009;325: 1660 doi: 10.1126/science.1178120 19779192 10.1126/science.1178120

[14] Righton DA, Westerberg H, Feunteun E, Okland F, Gargan P, Amilhat E, et al Empirical observations of the spawning migration of European eels: The long and dangerous road to the Sargasso Sea. Sci Adv. 2016;2: e1501694 doi: 10.1126/sciadv.1501694 27713924 10.1126/sciadv.1501694 PMC5052013

[15] Schabetsberger R, Miller MJ, Dall’Olmo G, Kaiser R, Økland F, Watanabe S, et al Hydrographic features of anguillid spawning areas: potential signposts for migrating eels. Mar Ecol Prog Ser. 2016;554: 141–155. doi: 10.3354/meps11824 10.3354/meps11824 PMC7116345 33184524

[16] Fänge R. Gas exchange in fish swim bladder. Rev Physiol Biochem Pharmacol. 1983;97: 111–158. Available: http://www.ncbi.nlm.nih.gov/pubmed/6408725 6408725 10.1007/BFb0035347

[17] Pelster B. Buoyancy at Depth In: Randall DJ, Farrel AP, editors. Deep Sea Fishes. San Diego, USA: Academic Press; 1997 pp. 195–237.

[18] Pelster B. The swimbladder In: Trischitta F, Takei Y, Sebert P, editors. Eel Physiology. Boca Raton, FL: CRC Press; 2013 pp. 44–67.

[19] Pelster B. Swimbladder function and the spawning migration of the European eel *Anguilla anguilla*. Front Physiol. 2015;5: 1–10. doi: 10.3389/fphys.2014.00486 25646080 10.3389/fphys.2014.00486 PMC4297919

[20] Tesch FW, Bartsch P, Berg R, Gabriel O, Henderson IW, Kamstra A, et al The Eel. 3rd ed Thorpe JE, editor. Oxford, UK: Blackwell Science Ltd; 2003.

[21] van Ginneken VJT, Maes GE. The European eel (*Anguilla anguilla*, Linnaeus), its Lifecycle, Evolution and Reproduction: A Literature Review. Rev Fish Biol Fish. 2005;15: 367–398. doi: 10.1007/s11160-006-0005-8

[22] Righton DA, Aarestrup K, Jellyman D, Sébert P, van den Thillart GEEJM, Tsukamoto K. The *Anguilla* spp. migration problem: 40 million years of evolution and two millennia of speculation. J Fish Biol. 2012;81: 365–386. doi: 10.1111/j.1095-8649.2012.03373.x 22803715 10.1111/j.1095-8649.2012.03373.x

[23] Kleckner RC. Swim bladder volume maintenance related to initial oceanic migratory depth in silver-phase *Anguilla rostrata*. Science. 1980;208: 1481–1482. Available: http://www.ncbi.nlm.nih.gov/pubmed/7384792 7384792 10.1126/science.7384792

[24] Kleckner RC. Swimbladder wall guanine enhancement related to migratory depth in silver phase *Anguilla rostrata*. Comp Biochem Physiol Part A Physiol. 1980;65: 351–354. doi: 10.1016/0300-9629(80)90041-9

[25] Yamada Y, Zhang H, Okamura A, Tanaka S, Horie N, Mikawa N, et al Morphological and histological changes in the swim bladder during maturation of the Japanese eel. J Fish Biol. 2001;58: 804–814. doi: 10.1006/jfbi.2000.1486

[26] Pelster B, Schneebauer G, Dirks RP. *Anguillicola* crassus Infection Significantly Affects the Silvering Related Modifications in Steady State mRNA Levels in Gas Gland Tissue of the European Eel. Front Physiol. 2016;7: 1–13. 27242549 10.3389/fphys.2016.00175 PMC4876612

[27] Kirk RS. The impact of *Anguillicola crassus* on European eels. Fish Manag Ecol. 2003;10: 385–394. doi: 10.1111/j.1365-2400.2003.00355.x

[28] Lefebvre F, Fazio G, Mounaix B, Crivelli AJ. Is the continental life of the European eel *Anguilla anguilla* affected by the parasitic invader *Anguillicoloides crassus*? Proc R Soc B Biol Sci. 2013;280: 20122916. doi: 10.1098/rspb.2012.2916 23325776 10.1098/rspb.2012.2916 PMC3574337

[29] Würtz J, Taraschewski H, Pelster B. Changes in gas composition in the swimbladder of the European eel (*Anguilla anguilla*) infected with *Anguillicola crassus* (Nematoda). Parasitology. 1996;112: 233–238. Available: http://www.ncbi.nlm.nih.gov/pubmed/8851864 8851864 10.1017/s003118200008481x

[30] Würtz J, Taraschewski H. Histopathological changes in the swimbladder wall of the European eel *Anguilla anguilla* due to infections with *Anguillicola crassus*. Dis Aquat Organ. 2000;39: 121–134. doi: 10.3354/dao039121 10715817 10.3354/dao039121

[31] Barry J, McLeish J, Dodd JA, Turnbull JF, Boylan P, Adams CE. Introduced parasite Anguillicola crassus infection significantly impedes swim bladder function in the European eel Anguilla anguilla (L.). J Fish Dis. 2014;37: 921–924. doi: 10.1111/jfd.12215 24422641 10.1111/jfd.12215

[32] Schneebauer G, Hanel R, Pelster B. *Anguillicola crassus* impairs the silvering-related enhancements of the ROS defense capacity in swimbladder tissue of the European eel (*Anguilla anguilla*). J Comp Physiol B. Springer Berlin Heidelberg; 2016;186: 867–877. doi: 10.1007/s00360-016-0994-0 27146148 10.1007/s00360-016-0994-0 PMC5009179

[33] Fazio G, Sasal P, Mouahid G, Lecomte-Finiger R, Moné H. Swim bladder nematodes (Anguillicoloides crassus) disturb silvering in European eels (Anguilla anguilla). J Parasitol. 2012;98: 695–705. doi: 10.1645/GE-2700.1 22404329 10.1645/GE-2700.1

[34] Morris SM, Albright JT. Superoxide dismutase, catalase, and glutathione peroxidase in the swim bladder of the physoclistous fish, Opsanus tau L. Cell Tissue Res. 1981;220: 739–752. Available: http://www.ncbi.nlm.nih.gov/pubmed/7296650 7296650 10.1007/BF00210458

[35] Morris SM, Albright JT. Catalase, glutathione peroxidase, and superoxide dismutase in the rete mirabile and gas gland epithelium of six species of marine fishes. J Exp Zool. 1984;232: 29–39. doi: 10.1002/jez.1402320105 6502092 10.1002/jez.1402320105

[36] Lushchak VI, Semchyshyn HM. Oxidative stress-Molecular mechanisms and Biological effects. Lushchak V, Halyna MS, editors. Rijeka, Croatia: InTech; 2012.

[37] Lefebvre F, Fazio G, Palstra AP, Székely C, Crivelli AJ. An evaluation of indices of gross pathology associated with the nematode Anguillicoloides crassus in eels. J Fish Dis. 2011;34: 31–45. doi: 10.1111/j.1365-2761.2010.01207.x 21118268 10.1111/j.1365-2761.2010.01207.x

[38] Dufour S, Burzawa-Gerard E, Le Belle N, Sbaihi M, Vidal B. Reproductive endocrinology of the European eel, Anguilla anguilla In: Aida K, Sukamoto K, Yamauchi K, editors. Eel Biology. Tokio: Springer Japan; 2003 pp. 373–383.

[39] Als TD, Hansen MM, Maes GE, Castonguay M, Riemann L, Aarestrup K, et al All roads lead to home: panmixia of European eel in the Sargasso Sea. Mol Ecol. 2011;20: 1333–1346. doi: 10.1111/j.1365-294X.2011.05011.x 21299662 10.1111/j.1365-294X.2011.05011.x

[40] Pujolar JM, Jacobsen MW, Als TD, Frydenberg J, Munch K, Jónsson B, et al Genome-wide single-generation signatures of local selection in the panmictic European eel. Mol Ecol. 2014;23: 2514–2528. doi: 10.1111/mec.12753 24750353 10.1111/mec.12753

[41] Durif CMF, Dufour S, Elie P. The silvering process of Anguilla anguilla: a new classification from the yellow resident to the silver migrating stage. J Fish Biol. 2005;66: 1025–1043.

[42] Pankhurst NW. Relation of visual changes to the onset of sexual maturation in the European eel Anguilla anguilla (L.). J Fish Biol. 1982;21: 127–140. doi: 10.1111/j.1095-8649.1982.tb03994.x

[43] Dirks RP, Burgerhout E, Brittijn SA, de Wijze DL, Ozupek H, Tuinhof-Koelma N, et al Identification of molecular markers in pectoral fin to predict artificial maturation of female European eels (Anguilla anguilla). Gen Comp Endocrinol. Elsevier Inc.; 2014;204: 267–276. doi: 10.1016/j.ygcen.2014.06.023 24992558 10.1016/j.ygcen.2014.06.023

[44] Burgerhout E, Minegishi Y, Brittijn SA, de Wijze DL, Henkel C V, Jansen HJ, et al Changes in ovarian gene expression profiles and plasma hormone levels in maturing European eel (Anguilla anguilla); Biomarkers for broodstock selection. Gen Comp Endocrinol. Elsevier Inc.; 2016;225: 185–196. doi: 10.1016/j.ygcen.2015.08.006 26255685 10.1016/j.ygcen.2015.08.006

[45] Henkel C V, Burgerhout E, de Wijze DL, Dirks RP, Minegishi Y, Jansen HJ, et al Primitive duplicate Hox clusters in the European eel’s genome. PLoS One. 2012;7: e32231 doi: 10.1371/journal.pone.0032231 22384188 10.1371/journal.pone.0032231 PMC3286462

[46] Trapnell C, Pachter L, Salzberg SL. TopHat: discovering splice junctions with RNA-Seq. Bioinformatics. 2009;25: 1105–1111. doi: 10.1093/bioinformatics/btp120 19289445 10.1093/bioinformatics/btp120 PMC2672628

[47] Li H, Handsaker B, Wysoker A, Fennell T, Ruan J, Homer N, et al The Sequence Alignment/Map format and SAMtools. Bioinformatics. 2009;25: 2078–2079. doi: 10.1093/bioinformatics/btp352 19505943 10.1093/bioinformatics/btp352 PMC2723002

[48] Anders S, Pyl PT, Huber W. HTSeq—a Python framework to work with high-throughput sequencing data. Bioinformatics. 2015;31: 166–169. doi: 10.1093/bioinformatics/btu638 25260700 10.1093/bioinformatics/btu638 PMC4287950

[49] Anders S, Huber W. Differential expression analysis for sequence count data. Genome Biol. 2010;11: R106 doi: 10.1186/gb-2010-11-10-r106 20979621 10.1186/gb-2010-11-10-r106 PMC3218662

[50] van Banning P, Haenen OLM. Effects of the swimbladder nematode *Anguillicola crassus* in wild and farmed eel, *Anguilla anguilla* Pathology in Marine Science. Elsevier; 1990 pp. 317–330. doi: 10.1016/B978-0-12-550755-4.50037-9

[51] Nimeth K, Zwerger P, Würtz J, Salvenmoser W, Pelster B. Infection of the glass-eel swimbladder with the nematode *Anguillicola crassus*. Parasitology. 2000;121: 75–83. Available: http://www.ncbi.nlm.nih.gov/pubmed/11085227 11085227 10.1017/s003118209900606x

[52] Beregi A, Molnár K, Békési L, Székely C. Radiodiagnostic method for studying swimbladder inflammation caused by *Anguillicola crassus* (Nematoda: Dracunculoidea). Dis Aquat Organ. 1998;34: 155–160. doi: 10.3354/dao034155 9828409 10.3354/dao034155

[53] Würtz J, Knopf K, Taraschewski H. Distribution and prevalence of *Anguillicola crassus* (Nematoda) in eels *Anguilla anguilla* of the rivers Rhine and Naab, Germany. Dis Aquat Organ. 1998;32: 137–143. doi: 10.3354/dao032137 9676253 10.3354/dao032137

[54] Knopf K, Madriles Helm A, Lucius R, Bleiss W, Taraschewski H. Migratory response of European eel (*Anguilla anguilla*) phagocytes to the eel swimbladder nematode *Anguillicola crassus*. Parasitol Res. 2008;102: 1311–1316. doi: 10.1007/s00436-008-0910-y 18311570 10.1007/s00436-008-0910-y

[55] Knopf K, Naser K, van der Heijden MHT, Taraschewski H. Humoral immune response of European eel *Anguilla anguilla* experimentally infected with *Anguillicola crassus*. Dis Aquat Organ. 2000;42: 61–69. doi: 10.3354/dao042061 10986646 10.3354/dao042061

[56] Kennedy CR. The pathogenic helminth parasites of eels. J Fish Dis. 2007;30: 319–334. doi: 10.1111/j.1365-2761.2007.00821.x 17498176 10.1111/j.1365-2761.2007.00821.x

[57] Zhu Z, Zheng T, Homer RJ, Kim Y-K, Chen NY, Cohn L, et al Acidic Mammalian Chitinase in Asthmatic Th2 Inflammation and IL-13 Pathway Activation. Science. 2004;304: 1678–1682. doi: 10.1126/science.1095336 15192232 10.1126/science.1095336

[58] Wang X, Yu YY, Lieu S, Yang F, Lang J, Lu C, et al MMP9 regulates the cellular response to inflammation after skeletal injury. Bone. 2013;52: 111–119. doi: 10.1016/j.bone.2012.09.018 23010105 10.1016/j.bone.2012.09.018 PMC3513654

[59] Schabuss M, Kennedy CR, Konecny R, Grillitsch B, Reckendorfer W, Schiemer F, et al Dynamics and predicted decline of *Anguillicola crassus* infection in European eels, *Anguilla anguilla*, in Neusiedler See, Austria. J Helminthol. 2005;79: 159–167. doi: 10.1079/JOH2005281 15946398 10.1079/joh2005281

[60] Clow KA, Short CE, Hall JR, Gendron RL, Driedzic WR. High rates of glucose utilization in the gas gland of Atlantic cod (*Gadus morhua*) are supported by GLUT1 and HK1b. J Exp Biol. 2016;219: 2763–2773. doi: 10.1242/jeb.141721 27401755 10.1242/jeb.141721

[61] Lemire JM, Braun KR, Maurel P, Kaplan ED, Schwartz SM, Wight TN. Versican/PG-M Isoforms in Vascular Smooth Muscle Cells. Arterioscler Thromb Vasc Biol. 1999;19: 1630–1639. doi: 10.1161/01.ATV.19.7.1630 10397680 10.1161/01.atv.19.7.1630

[62] Morris AH, Kyriakides TR. Matricellular proteins and biomaterials. Matrix Biol. International Society of Matrix Biology; 2014;37: 183–191. doi: 10.1016/j.matbio.2014.03.002 24657843 10.1016/j.matbio.2014.03.002 PMC4167162

[63] Kim I, Moon S-O, Koh KN, Kim H, Uhm C-S, Kwak HJ, et al Molecular Cloning, Expression, and Characterization of Angiopoietin-related Protein: Angiopoietin-Related Protein Induces Endothelial Cell Sprouting. J Biol Chem. 1999;274: 26523–26528. doi: 10.1074/jbc.274.37.26523 10473614 10.1074/jbc.274.37.26523

[64] Pelster B. Mechanisms of acid release in isolated gas gland cells of the European eel *Anguilla anguilla*. Am J Physiol. 1995;269: R793–R799. Available: http://www.ncbi.nlm.nih.gov/pubmed/7485595 7485595 10.1152/ajpregu.1995.269.4.R793

[65] Pelster B, Niederstätter H. pH-dependent proton secretion in cultured swim bladder gas gland cells. Am J Physiol. 1997;273: R1719–R1725. Available: http://www.ncbi.nlm.nih.gov/pubmed/9374815 9374815 10.1152/ajpregu.1997.273.5.R1719

[66] Pelster B. pH regulation and swimbladder function in fish. Respir Physiol Neurobiol. 2004;144: 179–190. doi: 10.1016/j.resp.2004.03.019 15556101 10.1016/j.resp.2004.03.019

[67] Stepniak E. c-Jun/AP-1 controls liver regeneration by repressing p53/p21 and p38 MAPK activity. Genes Dev. 2006;20: 2306–2314. doi: 10.1101/gad.390506 16912279 10.1101/gad.390506 PMC1553212

[68] Neub A, Houdek P, Ohnemus U, Moll I, Brandner JM. Biphasic Regulation of AP-1 Subunits during Human Epidermal Wound Healing. J Invest Dermatol. Elsevier Masson SAS; 2007;127: 2453–2462. doi: 10.1038/sj.jid.5700864 17495958 10.1038/sj.jid.5700864

[69] Shaulian E. AP-1—The Jun proteins: Oncogenes or tumor suppressors in disguise? Cell Signal. Elsevier Inc.; 2010;22: 894–899. doi: 10.1016/j.cellsig.2009.12.008 20060892 10.1016/j.cellsig.2009.12.008

[70] Dinauer MC, Pierce EA, Bruns GAP, Curnutte JT, Orkin SH. Human neutrophil cytochrome b light chain (p22-phox). Gene structure, chromosomal location, and mutations in cytochrome-negative autosomal recessive chronic granulomatous disease. J Clin Invest. 1990;86: 1729–1737. doi: 10.1172/JCI114898 2243141 10.1172/JCI114898 PMC296926

[71] Guengerich FP. Reactions and significance of cytochrome P-450 enzymes. J Biol Chem. 1991;266: 10019–10022. Available: http://www.ncbi.nlm.nih.gov/pubmed/2037557 2037557

[72] Lek A, Evesson FJ, Sutton RB, North KN, Cooper ST. Ferlins: Regulators of Vesicle Fusion for Auditory Neurotransmission, Receptor Trafficking and Membrane Repair. Traffic. 2012;13: 185–194. doi: 10.1111/j.1600-0854.2011.01267.x 21838746 10.1111/j.1600-0854.2011.01267.x

[73] Yáñez M, Gil-Longo J, Campos-Toimil M. Calcium Binding Proteins In: Islam MS, editor. Calcium Signaling. Dordrecht: Springer Netherlands; 2012 pp. 461–482. doi: 10.1007/978-94-007-2888-2_19 10.1007/978-94-007-2888-2_19 22453954

[74] Sébert P, Vettier A, Moisan C. High pressure resistance and adaptation of European eels In: van den Thillart G, Dufour S, Rankin JC, editors. Spawning Migration of the European Eel. New York: Springer Science; 2009 pp. 99–127.

[75] van Ginneken VJT, Durif CMF, Balm SP, Boot R, Verstegen M, Antonissen E, et al Silvering of European eel (*Anguilla anguilla L*.): seasonal changes of morphological and metabolic parameters. Anim Biol. 2007;57: 63–77. doi: 10.1163/157075607780002014

[76] Wysujack K, Westerberg H, Aarestrup K, Trautner J, Kurwie T, Nagel F, et al The migration behaviour of European silver eels (*Anguilla anguilla*) released in open ocean conditions. Mar Freshw Res. 2015;66: 145–157. doi: 10.1071/MF14023

