# Supplementary material for: Anguillicola crassus infection affects mRNA expression levels in gas gland tissue of European yellow and silver eel
Source: PLoS One. 2017 Aug 17;12(8):e0183128. doi: 10.1371/journal.pone.0183128 (PMC5560681; doi:10.1371/journal.pone.0183128)
Supplement: S1 Table — (DOCX) [file pone.0183128.s001.docx]

| **Gene** | **Name** | **Description** | **Yellow** | | **Silver** | |
| --- | --- | --- | --- | --- | --- | --- |
|  |  |  | **Fold cha.** | **pval** | **Fold cha.** | **pval** |
| g42062 | sc5a8 | sodium-coupled monocarboxylate transporter 1 | Inf | 0.000 |  |  |
| g14954 | sc5a8 | sodium-coupled monocarboxylate transporter 1 | 18.00 | 0.002 |  |  |
| g38820 | gima1 | gtpase imap family member 1 | 12.32 | 0.008 |  |  |
| g5236 | s6a15 | orphan sodium- and chloride-dependent neurotransmitter transporter ntt73 | 11.49 | 0.000 |  |  |
| g10399 | plcz1 | 1-phosphatidylinositol- -bisphosphate phosphodiesterase zeta-1 | 8.85 | 0.003 |  |  |
| g24556 | gpr35 | g-protein coupled receptor 35 | 8.76 | 0.000 |  |  |
| g30502 | gima4 | gtpase imap family member 4 | 8.37 | 0.000 |  |  |
| g6900 | nfac2 | nuclear factor of activated t- cytoplasmic 2 | 5.46 | 0.000 |  |  |
| g3654 | pk2l1 | polycystic kidney disease 2-like 1 protein | 5.31 | 0.000 |  |  |
| g24053 | gima4 | gtpase imap family member 4 | 5.21 | 0.000 |  |  |
| g3764 | ctgf | connective tissue growth factor | 5.14 | 0.000 |  |  |
| g12086 | l3bpb | galectin-3-binding protein b | 5.06 | 0.000 |  |  |
| g40561 | vdac2 | voltage-dependent anion-selective channel protein 2 | 5.04 | 0.000 |  |  |
| g10121 | lck | tyrosine-protein kinase lck | 4.67 | 0.000 |  |  |
| g17339 | cxcr4 | c-x-c chemokine receptor type 4 | 4.66 | 0.000 |  |  |
| g14635 | gima7 | gtpase imap family member 7 | 4.44 | 0.000 |  |  |
| g6522 | trpa1 | transient receptor potential cation channel subfamily a member 1 | 4.13 | 0.005 |  |  |
| g40915 | gima4 | gtpase imap family member 4 | 4.12 | 0.000 |  |  |
| g4725 | cyr61 | protein cyr61 | 4.00 | 0.000 |  |  |
| g9327 | il18r | interleukin-18 receptor 1 | 3.99 | 0.006 |  |  |
| g18814 | ylat2 | y+l amino acid transporter 2 | 3.91 | 0.000 |  |  |
| g18935 | p2ry4 | p2y purinoceptor 4 | 3.82 | 0.004 |  |  |
| g3335 | p2y14 | p2y purinoceptor 14 | 3.81 | 0.003 |  |  |
| g3453 | s6a19 | sodium-dependent neutral amino acid transporter b at1 | 3.76 | 0.000 |  |  |
| g9191 | s12a2 | solute carrier family 12 member 2 | 3.67 | 0.001 |  |  |
| g27889 | fyn | tyrosine-protein kinase fyn | 3.46 | 0.000 |  |  |
| g21625 | trpc4 | short transient receptor potential channel 4 | 3.42 | 0.004 |  |  |
| g42262 | s4a4 | electrogenic sodium bicarbonate cotransporter 1 | 3.38 | 0.000 |  |  |
| g39226 | gima4 | gtpase imap family member 4 | 3.38 | 0.000 |  |  |
| g12497 | kpcb | protein kinase c beta type | 3.34 | 0.004 |  |  |
| g35485 | gima7 | gtpase imap family member 7 | 3.29 | 0.000 |  |  |
| g4039 | cdk1 | cell division protein kinase 1 | 3.19 | 0.000 |  |  |
| g12469 | kcnc1 | potassium voltage-gated channel subfamily c member 1 | 3.09 | 0.003 |  |  |
| g6321 | hphl1 | hephaestin-like protein 1 flags: precursor | 3.05 | 0.000 |  |  |
| g26034 | cp27a | sterol 26- mitochondrial | 0.32 | 0.000 |  |  |
| g19141 | clcn2 | chloride channel protein 2 | 0.26 | 0.001 |  |  |
| g17084 | achb2 | neuronal acetylcholine receptor subunit beta-2 | 0.24 | 0.005 |  |  |
| g30946 | gima7 | gtpase imap family member 7 | 0.17 | 0.000 |  |  |
| g21400 | kcma1 | calcium-activated potassium channel subunit alpha-1 | 0.17 | 0.000 |  |  |
| g35977 | sax | saxiphilin short=sax flags: precursor | 0.16 | 0.000 |  |  |
| g43801 | pp2ba | serine threonine-protein phosphatase 2b catalytic subunit alpha isoform | 0.15 | 0.000 |  |  |
| g3822 | cng1 | cyclic nucleotide-gated channel cone photoreceptor subunit alpha | 0.12 | 0.002 |  |  |
| g7841 | at1b2 | sodium potassium-transporting atpase subunit beta-2 | 0.12 | 0.000 |  |  |
| g21922 | s12a5 | solute carrier family 12 member 5 | 0.10 | 0.004 |  |  |
| g3113 | hxk4 | glucokinase | 0.08 | 0.009 |  |  |
| g25966 | gima5 | gtpase imap family member 5 | 0.05 | 0.003 |  |  |
| g649 | cftr | cystic fibrosis transmembrane conductance regulator | 0.05 | 0.000 |  |  |
| g7353 | so2a1 | solute carrier organic anion transporter family member 2a1 | 0.04 | 0.000 |  |  |
| g9182 | scn5a | sodium channel protein type 5 subunit alpha | 0.03 | 0.000 |  |  |
| g17469 | accn1 | amiloride-sensitive cation channel neuronal | 0.00 | 0.000 |  |  |
| g5564 | s39ac | zinc transporter zip12 | 7.17 | 0.000 | 0.22 | 0.006 |
| g11761 | s13a3 | solute carrier family 13 member 3 | 4.82 | 0.000 | 8.84 | 0.001 |
| g18418 | sc5a1 | sodium glucose cotransporter 1 | 4.41 | 0.000 | 31.70 | 0.000 |
| g3715 | lpar6 | lysophosphatidic acid receptor 6 | 4.02 | 0.002 | 6.88 | 0.000 |
| g16142 | ticn1 | testican-1 | 0.21 | 0.000 | 16.89 | 0.000 |
| g23142 | hecw1 | e3 ubiquitin-protein ligase hecw1 |  |  | Inf | 0.002 |
| g16672 | smoc1 | sparc-related modular calcium-binding protein 1 |  |  | Inf | 0.003 |
| g26738 | hfe | hereditary hemochromatosis protein |  |  | 24.64 | 0.000 |
| g34977 | ffar2 | free fatty acid receptor 2 |  |  | 20.05 | 0.000 |
| g16557 | s6a14 | sodium- and chloride-dependent neutral and basic amino acid transporter b(0+) |  |  | 16.13 | 0.000 |
| g11255 | wisp3 | wnt1-inducible-signaling pathway protein 3 |  |  | 9.00 | 0.003 |
| g7770 | mt12b | monocarboxylate transporter 12-b |  |  | 4.91 | 0.006 |
| g23306 | gima4 | gtpase imap family member 4 |  |  | 3.78 | 0.007 |
| g2350 | eaa2 | excitatory amino acid transporter 2 |  |  | 3.24 | 0.001 |
| g15264 | acc2a | amiloride-sensitive cation channel 2- neuronal |  |  | 0.26 | 0.009 |
| g14408 | p3 | p3 protein |  |  | 0.11 | 0.000 |
| g38167 | p3 | p3 protein |  |  | 0.11 | 0.000 |
| g25543 | gima7 | gtpase imap family member 7 |  |  | 0.10 | 0.000 |
| g17810 | atng | sodium potassium-transporting atpase subunit gamma |  |  | 0.05 | 0.000 |

Fold cha. = Fold change; pval = 0.000 indicates P values < 0.0005
