# Supplementary material for: Anguillicola crassus infection affects mRNA expression levels in gas gland tissue of European yellow and silver eel
Source: PLoS One. 2017 Aug 17;12(8):e0183128. doi: 10.1371/journal.pone.0183128 (PMC5560681; doi:10.1371/journal.pone.0183128)
Supplement: S2 Table — (DOCX) [file pone.0183128.s002.docx]

| **Gene** | **Name** | **Description** | **Yellow** | | **Silver** | |
| --- | --- | --- | --- | --- | --- | --- |
|  |  |  | **Fold cha.** | **pval** | **Fold cha.** | **pval** |
| g38341 | angl7 | angiopoietin-related protein 7 | Inf | 0.004 |  |  |
| g3270 | tr11b | tumor necrosis factor receptor superfamily member 11b | Inf | 0.009 |  |  |
| g11898 | fosb | protein fosb | 21.55 | 0.000 |  |  |
| g12410 | fos | proto-oncogene c-fos | 20.06 | 0.000 |  |  |
| g4495 | dnjb5 | homolog subfamily b member 5 | 10.83 | 0.000 |  |  |
| g5407 | hspbb | heat shock protein beta-11 | 10.00 | 0.000 |  |  |
| g20320 | morc3 | morc family cw-type zinc finger protein 3 | 8.99 | 0.003 |  |  |
| g7571 | ccna2 | cyclin-a2 | 7.62 | 0.000 |  |  |
| g9306 | dnjb4 | homolog subfamily b member 4 | 6.64 | 0.000 |  |  |
| g1691 | tsp1 | thrombospondin-1 flags: precursor | 6.46 | 0.000 |  |  |
| g16469 | tri69 | tripartite motif-containing protein 69 | 6.12 | 0.000 |  |  |
| g21129 | aff3 | af4 fmr2 family member 3 | 5.96 | 0.000 |  |  |
| g2441 | rgs8 | regulator of g-protein signaling 8 | 5.78 | 0.000 |  |  |
| g27065 | avt | vasotocin receptor | 5.73 | 0.000 |  |  |
| g2311 | ccnb1 | g2 mitotic-specific cyclin-b1 | 5.58 | 0.000 |  |  |
| g4109 | k1c18 | type i cytoskeletal 18 | 5.22 | 0.000 |  |  |
| g5642 | dus2 | dual specificity protein phosphatase 2 | 5.15 | 0.000 |  |  |
| g3764 | ctgf | connective tissue growth factor | 5.14 | 0.000 |  |  |
| g13449 | gtr5 | solute carrier family facilitated glucose transporter member 5 | 5.12 | 0.000 |  |  |
| g1342 | dnja4 | homolog subfamily a member 4 | 4.99 | 0.000 |  |  |
| g7750 | tsp4b | thrombospondin-4-b | 4.97 | 0.000 |  |  |
| g12409 | fos | proto-oncogene c-fos | 4.96 | 0.000 |  |  |
| g26051 | zap70 | tyrosine-protein kinase zap-70 | 4.91 | 0.000 |  |  |
| g1568 | b4 | protein b4 | 4.36 | 0.002 |  |  |
| g21556 | st2b1 | sulfotransferase family cytosolic 2b member 1 | 4.18 | 0.000 |  |  |
| g6522 | trpa1 | transient receptor potential cation channel subfamily a member 1 | 4.13 | 0.005 |  |  |
| g7816 | hmox | heme oxygenase | 4.05 | 0.000 |  |  |
| g4725 | cyr61 | protein cyr61 | 4.00 | 0.000 |  |  |
| g2197 | bc11b | b-cell lymphoma leukemia 11b | 3.86 | 0.000 |  |  |
| g3322 | fos | proto-oncogene c-fos | 3.70 | 0.000 |  |  |
| g20676 | csf1r | macrophage colony-stimulating factor 1 receptor | 3.60 | 0.000 |  |  |
| g38019 | ksyk | tyrosine-protein kinase syk | 3.59 | 0.000 |  |  |
| g27889 | fyn | tyrosine-protein kinase fyn | 3.46 | 0.000 |  |  |
| g12291 | rac2 | ras-related c3 botulinum toxin substrate 2 | 3.42 | 0.000 |  |  |
| g5094 | scub3 | signal cub and egf-like domain-containing protein 3 flags: precursor | 3.38 | 0.010 |  |  |
| g15583 | nud17 | nucleoside diphosphate-linked moiety x motif 17 | 3.38 | 0.000 |  |  |
| g18110 | par11 | poly polymerase 11 | 3.36 | 0.002 |  |  |
| g17446 | paxi | Paxillin | 3.35 | 0.000 |  |  |
| g12497 | kpcb | protein kinase c beta type | 3.34 | 0.004 |  |  |
| g15582 | gp112 | probable g-protein coupled receptor 112 flags: precursor | 3.33 | 0.000 |  |  |
| g16269 | svep1 | von willebrand factor type egf and pentraxin domain-containing protein 1 | 3.33 | 0.006 |  |  |
| g10772 | prdm1 | pr domain zinc finger protein 1 | 3.32 | 0.001 |  |  |
| g10644 | arrd4 | arrestin domain-containing protein 4 | 3.26 | 0.000 |  |  |
| g19804 | a33 | zinc-binding protein a33 | 3.23 | 0.001 |  |  |
| g4039 | cdk1 | cell division protein kinase 1 | 3.19 | 0.000 |  |  |
| g14921 | ets1a | protein c-ets-1-a a | 3.11 | 0.003 |  |  |
| g27891 | rdh8 | retinol dehydrogenase 8 | 0.33 | 0.000 |  |  |
| g24544 | nr0b2 | nuclear receptor subfamily 0 group b member 2 | 0.32 | 0.006 |  |  |
| g17607 | tnks1 | tankyrase-1 | 0.32 | 0.004 |  |  |
| g26034 | cp27a | sterol 26- mitochondrial | 0.32 | 0.000 |  |  |
| g23774 | ppara | peroxisome proliferator-activated receptor alpha | 0.31 | 0.005 |  |  |
| g3029 | tri25 | e3 ubiquitin isg15 ligase trim25 | 0.29 | 0.000 |  |  |
| g14208 | r1441 | probable e3 ubiquitin-protein ligase rnf144a-a | 0.26 | 0.000 |  |  |
| g19787 | hp55 | hibernation-specific plasma protein hp-55 | 0.23 | 0.000 |  |  |
| g13055 | tmpsd | transmembrane protease serine 13 | 0.23 | 0.001 |  |  |
| g28779 | cadm3 | cell adhesion molecule 3 | 0.16 | 0.002 |  |  |
| g3822 | cng1 | cyclic nucleotide-gated channel cone photoreceptor subunit alpha | 0.12 | 0.002 |  |  |
| g25457 | pias4 | e3 sumo-protein ligase pias4 | 0.06 | 0.005 |  |  |
| g3911 | anr45 | ankyrin repeat domain-containing protein 45 | 0.05 | 0.001 |  |  |
| g649 | cftr | cystic fibrosis transmembrane conductance regulator | 0.05 | 0.000 |  |  |
| g7353 | so2a1 | solute carrier organic anion transporter family member 2a1 | 0.04 | 0.000 |  |  |
| g22125 | twhh | tiggy-winkle hedgehog protein | Inf | 0.000 | Inf | 0.000 |
| g8653 | fr1l4 | fer-1-like protein 4 | 25.45 | 0.000 | 11.07 | 0.000 |
| g30820 | fr1l4 | fer-1-like protein 4 | 16.43 | 0.000 | 37.39 | 0.000 |
| g14663 | cp1b1 | cytochrome p450 1b1 | 11.08 | 0.000 | 0.22 | 0.005 |
| g27029 | adam8 | disintegrin and metalloproteinase domain-containing protein 8 | 3.83 | 0.000 | 12.02 | 0.000 |
| g12711 | mmp9 | matrix metalloproteinase-9 | 3.64 | 0.000 | 23.32 | 0.000 |
| g23142 | hecw1 | e3 ubiquitin-protein ligase hecw1 |  |  | Inf | 0.002 |
| g13985 | tdrd1 | tudor domain-containing protein 1 |  |  | 20.37 | 0.000 |
| g26855 | celf3 | cugbp elav-like family member 3 |  |  | 18.40 | 0.005 |
| g16557 | s6a14 | sodium- and chloride-dependent neutral and basic amino acid transporter b(0+) |  |  | 16.13 | 0.000 |
| g11255 | wisp3 | wnt1-inducible-signaling pathway protein 3 |  |  | 9.00 | 0.003 |
| g8823 | hyalp | hyaluronidase ph-20 |  |  | 7.46 | 0.001 |
| g5914 | dmbt1 | deleted in malignant brain tumors 1 protein |  |  | 5.17 | 0.002 |
| g25479 | g3st1 | galactosylceramide sulfotransferase |  |  | 0.29 | 0.006 |
| g9834 | cadm1 | cell adhesion molecule 1 |  |  | 0.26 | 0.002 |
| g1090 | ileu | leukocyte elastase inhibitor |  |  | 0.22 | 0.002 |
| g15262 | dyn1 | dynamin-1 |  |  | 0.20 | 0.003 |
| g21703 | s22ag | solute carrier family 22 member 16 |  |  | 0.19 | 0.001 |
| g24694 | mmp17 | matrix metalloproteinase-17 |  |  | 0.08 | 0.001 |
| g17364 | tecta | alpha-tectorin flags: precursor |  |  | 0.04 | 0.000 |
| g1804 | ima1 | importin subunit alpha-1 |  |  | 0.02 | 0.004 |
| g1675 | sox3 | transcription factor sox-3 |  |  | 0.02 | 0.006 |
| g36576 | zp3 | zona pellucida sperm-binding protein 3 |  |  | 0.01 | 0.000 |
| g45297 | zp2 | zona pellucida sperm-binding protein 2 |  |  | 0.01 | 0.000 |
| g15786 | zp1 | zona pellucida sperm-binding protein 1 |  |  | 0.00 | 0.000 |

Fold cha. = Fold change; pval = 0.000 indicates P values < 0.0005
